# Supplementary figures and images for: Computational models to improve surveillance for cassava brown streak disease and minimize yield loss
Source: PLoS Comput Biol. 2020 Jul 2;16(7):e1007823. doi: 10.1371/journal.pcbi.1007823 (PMC7331984; doi:10.1371/journal.pcbi.1007823)

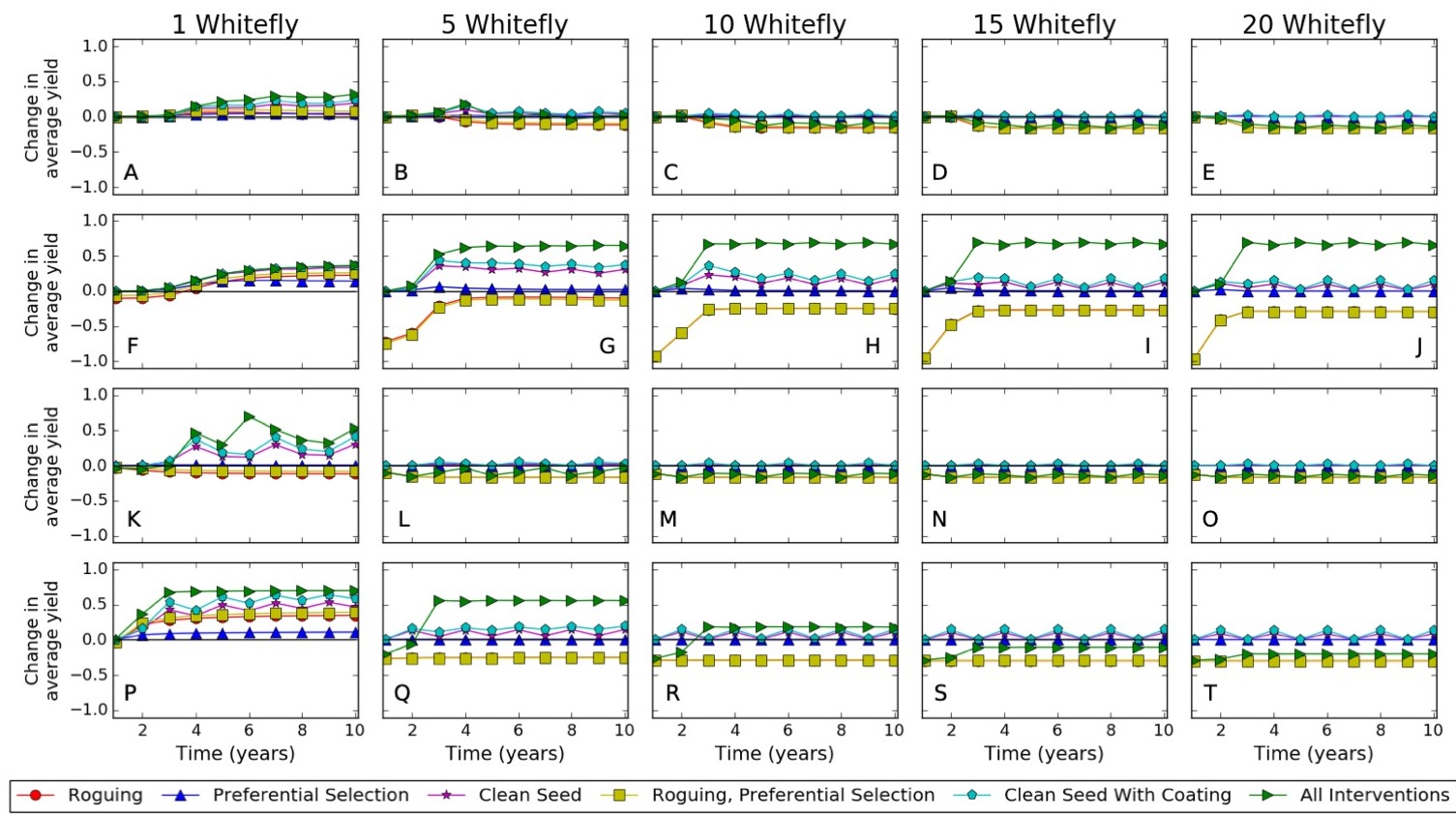

Supplement: S1 Fig — (A,B,C,D,E) start with one infected plant and use low intensity interventions, (F,G,H,I,J) start with one infected plant and use high intensity interventions, (K,L,M,N,O) start with a quarter of the field infected and use low intensity interventions, and (P,Q,R,S,T) start with a quarter of the field infected and use high intensity interventions. For each subplot, the x-axis is the number of seasons since the start of the epidemic. (TIF) [file pcbi.1007823.s001.tif]

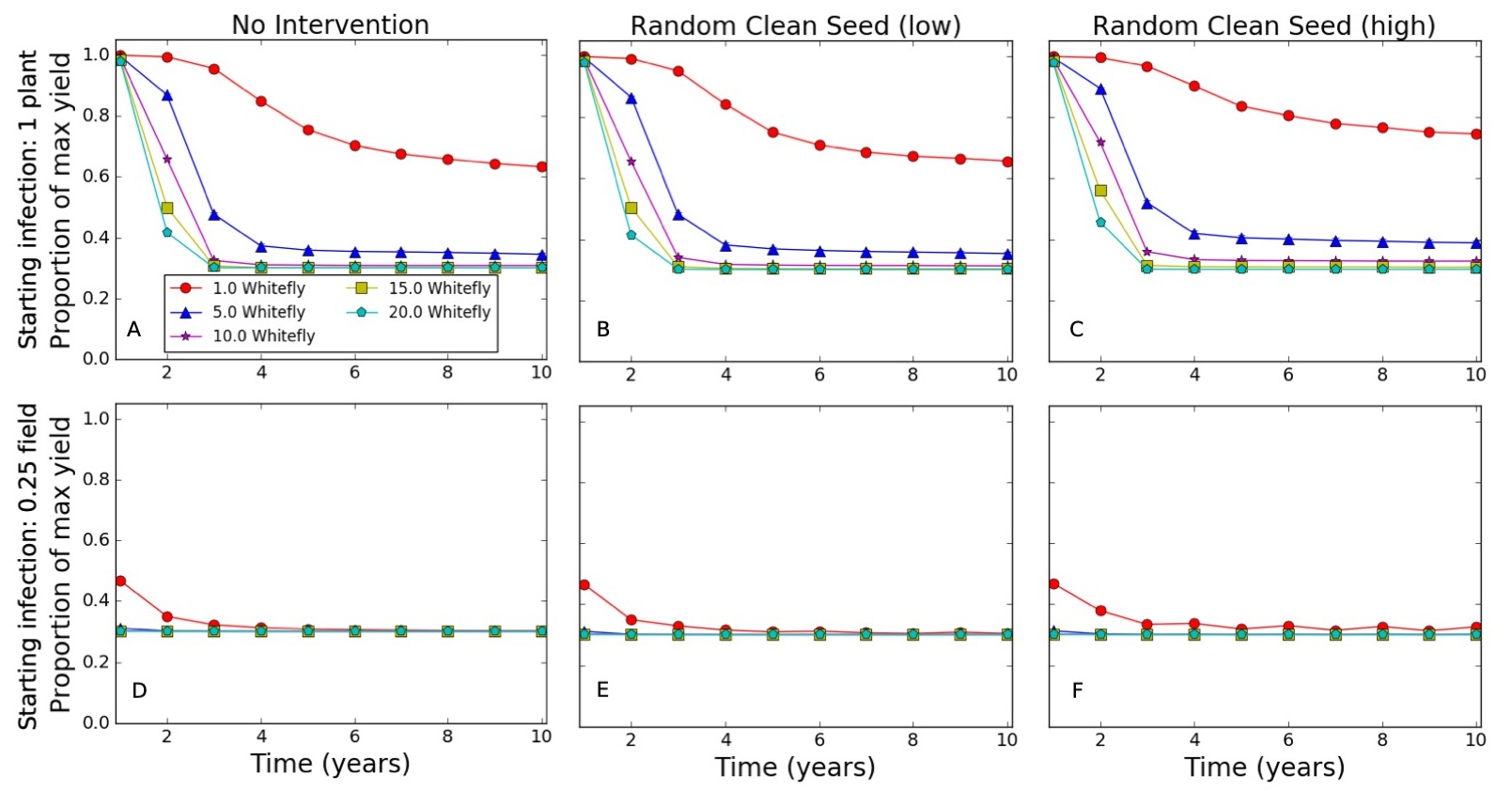

Supplement: S2 Fig — For each plot, the x-axis is the number of seasons since the start of the epidemic, and the y-axis is the average yield over an ensemble of 1000 epidemics for each management intervention. (A,B,C) have a starting infection of a single plant while (D,E,F) start with 25% of the field infected. (A,D) have no interventions, (B,E) use low intensity randomly planted clean seed, and (C,F) use high intensity randomly planted clean seed. (TIF) [file pcbi.1007823.s002.tif]

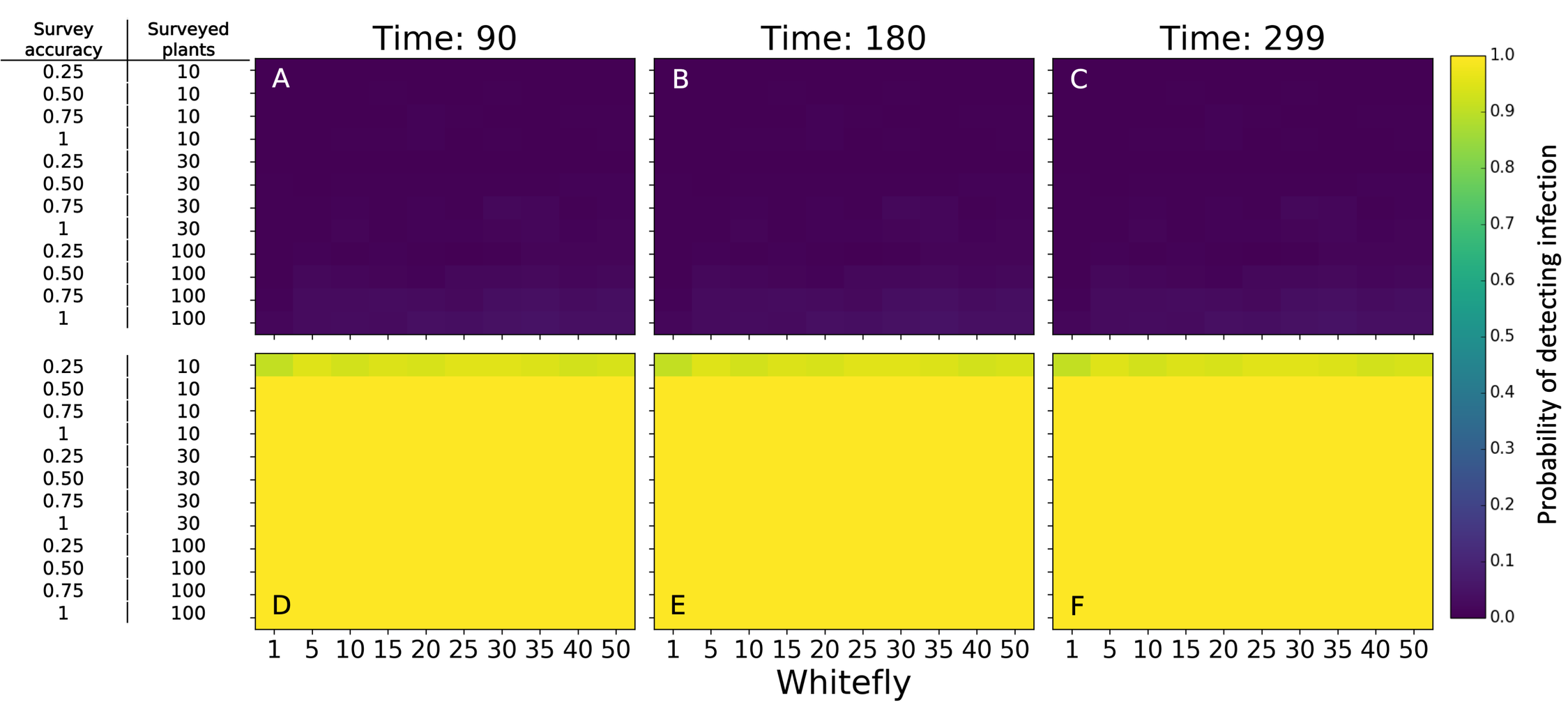

Supplement: S3 Fig — For each plot, the x-axis is the average number of whitefly per top five leaves on the plant, and the y-axis is a different combination of survey parameters (surveyor accuracy and number of plants surveyed). (A,B,C) have a starting infection of a single plant while (D,E,F) start with 20% of the field infected. The surveys were conducted at (A,D) 90 days after planting; (B,E) 180 days after planting; (C,F) 299 days after planting. (TIF) [file pcbi.1007823.s003.tif]

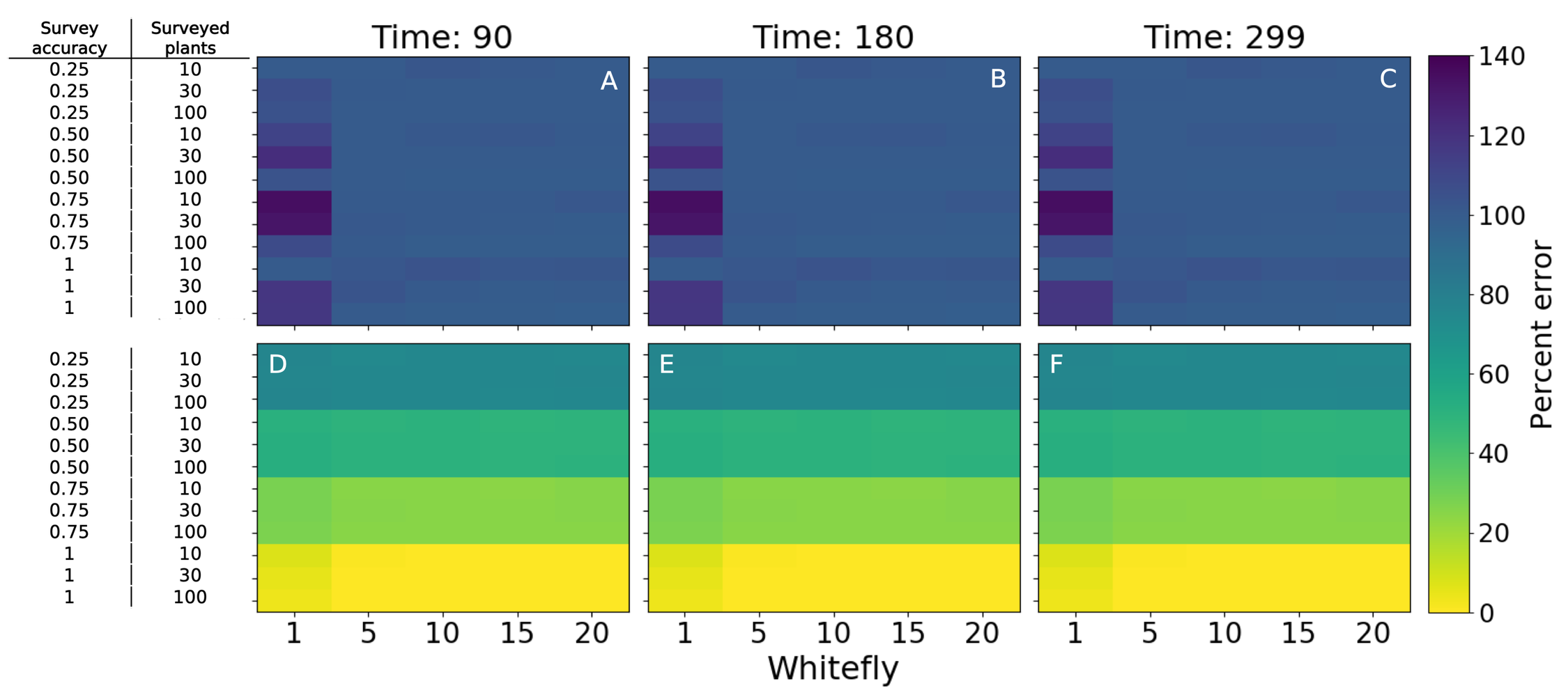

Supplement: S4 Fig — For each plot, the x-axis is the average number of whitefly per top five leaves on the plant, and the y-axis is a different combination of survey variables (surveyor accuracy and number of plants surveyed). (A,D) have surveys conducted 90 days after planting, (B,E) have surveys conducted 180 days after planting, and (C,F) have surveys conducted 299 days after planting. (A,B,C) have a starting infection of one plant and (D,E,F) have a starting infection of 50%. (TIF) [file pcbi.1007823.s004.tif]

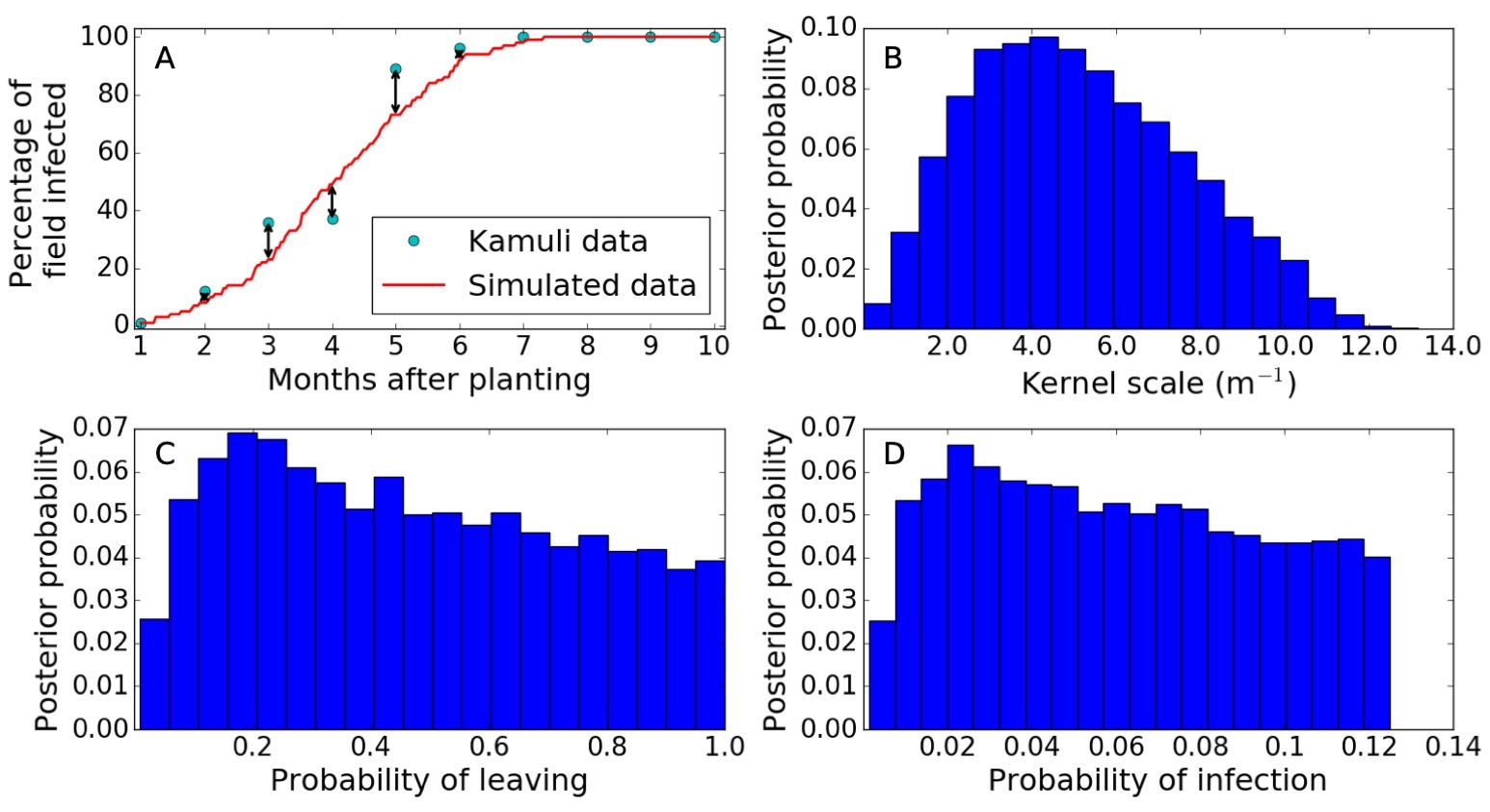

Supplement: S5 Fig — (A) shows experimental data from Katono et al. in cyan with an example of simulated data with an accepted set of parameters shown as a red line. Double headed arrows show the distance between the experimental and simulated data. (B,C,D) show marginal posterior probabilities for the parameter values. (TIF) [file pcbi.1007823.s005.tif]

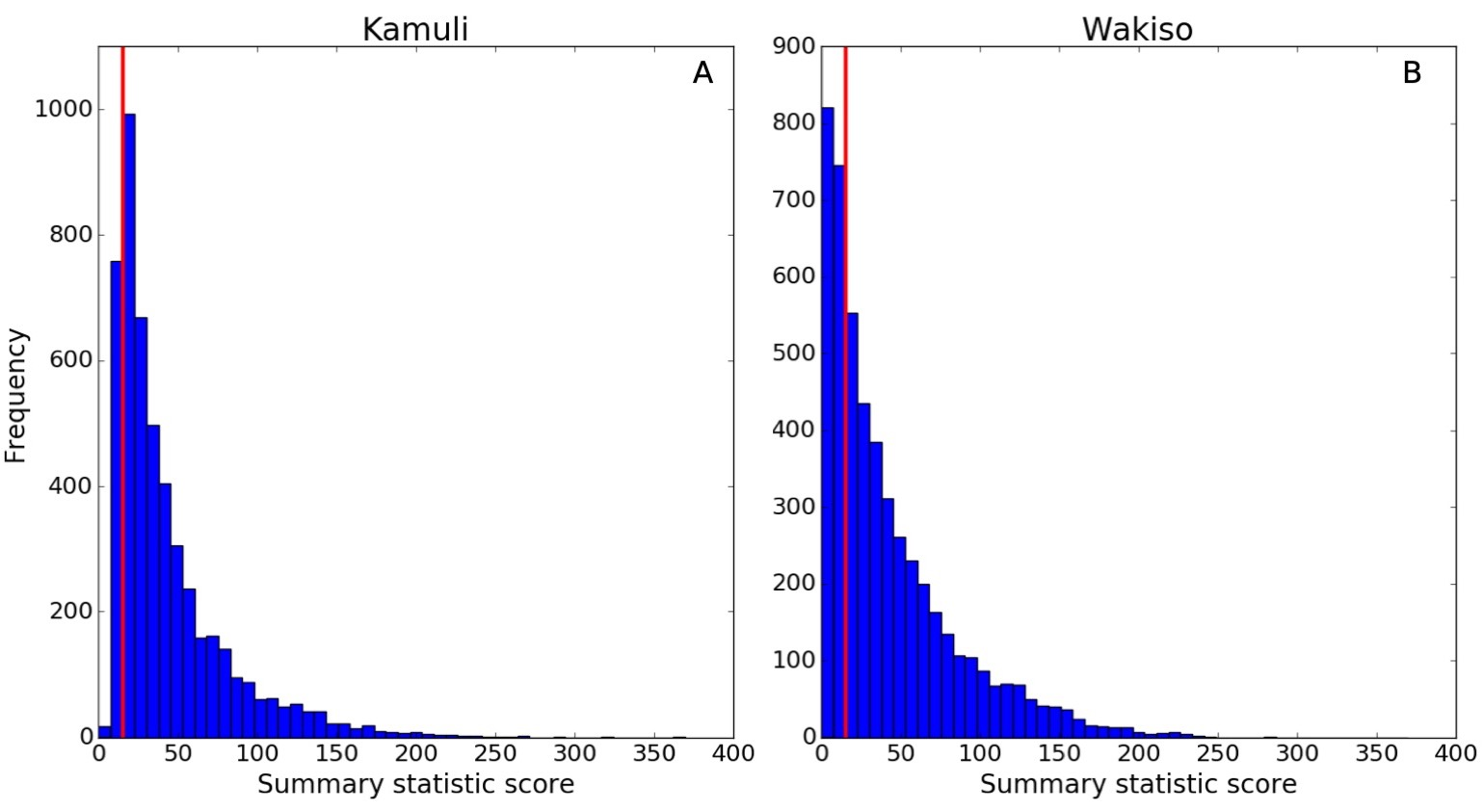

Supplement: S6 Fig — The red line indicates a summary statistic score of 15. Thirty one percent of the Wakiso simulations are below the cutoff value of 15 and 15% of the Kamuli simulations are below the cutoff. (TIF) [file pcbi.1007823.s006.tif]
