# Supplementary material for: Computational models to improve surveillance for cassava brown streak disease and minimize yield loss
Source: PLoS Comput Biol. 2020 Jul 2;16(7):e1007823. doi: 10.1371/journal.pcbi.1007823 (PMC7331984; doi:10.1371/journal.pcbi.1007823)
Supplement: Attachment — Submitted filename: PCOMPBIOL-D-19-01812_reviewer_06012020.pdf [file pcbi.1007823.s009.pdf]

PLOS Computational Biology

Computational models to improve surveillance for cassava brown streak disease and minimize yield loss.

--Manuscript Draft--

**Manuscript Number:**	PCOMPBIOL-D-19-01812
**Full Title:**	Computational models to improve surveillance for cassava brown streak disease and minimize yield loss.
**Short Title:**	Computational models to improve surveillance for CBSD and minimize yield loss.
**Article Type:**	Research Article
**Keywords:**	epidemiology modelling; cassava brown streak disease; surveillance; disease management
**Abstract:**	Cassava brown streak disease (CBSD) is a rapidly spreading viral disease that affects a major food security crop in sub-Saharan Africa. Currently, there are several proposed management interventions to minimize loss in infected fields. Field-scale data comparing the effectiveness of these interventions individually and in combination are limited and expensive to collect. Using a stochastic epidemiological model for the spread and management of CBSD in individual fields, we simulate the effectiveness of a range of management interventions. Specifically we compare the removal of diseased plants by roguing, preferential selection of planting material, deployment of virus-free 'clean seed' and pesticide on crop yield and disease status of individual fields with varying levels of whitefly density crops under low and high disease pressure. We examine management interventions for sustainable production of planting material in clean seed systems and how to improve survey protocols to identify the presence of CBSD in a field or quantify the within-field prevalence of CBSD. We also propose guidelines for practical, actionable recommendations for the deployment of management strategies in regions of sub-Saharan Africa under different disease and whitefly pressure.
**Additional Information:**	
**Question**	**Response**
**Financial Disclosure**<br><br>Enter a financial disclosure statement that describes the sources of funding for the work included in this submission. Review the submission guidelines for detailed requirements. View published research articles from *PLOS Computational Biology* for specific examples.<br><br>This statement is required for submission and **will appear in the published article** if the submission is accepted. Please make sure it is accurate.	CAG recieved a grant (RG67082) from the Bill and Melinda Gates Foundation (https://www.gatesfoundation.org). The funders had no role in study design, data collection and analysis, decision to publish, or preparation of the manuscript.

Powered by Editorial Manager® and ProduXion Manager® from Aries Systems Corporation

**Unfunded studies**<br>Enter: *The author(s) received no specific funding for this work.*<br><br>**Funded studies**<br>Enter a statement with the following details:<br>• Initials of the authors who received each award<br>• Grant numbers awarded to each author<br>• The full name of each funder<br>• URL of each funder website<br>• Did the sponsors or funders play any role in the study design, data collection and analysis, decision to publish, or preparation of the manuscript?<br>• **NO** - Include this sentence at the end of your statement: *The funders had no role in study design, data collection and analysis, decision to publish, or preparation of the manuscript.*<br>• **YES** - Specify the role(s) played.<br><br>* typeset	
**Competing Interests**<br><br>Use the instructions below to enter a competing interest statement for this submission. On behalf of all authors, disclose any competing interests that could be perceived to bias this work—acknowledging all financial support and any other relevant financial or non-financial competing interests.<br><br>This statement **will appear in the published article** if the submission is accepted. Please make sure it is accurate. View published research articles from *PLOS Computational Biology* for specific examples.	The authors have declared that no competing interests exist.

Powered by Editorial Manager® and ProduXion Manager® from Aries Systems Corporation

**NO authors have competing interests**<br><br>Enter: *The authors have declared that no competing interests exist.*<br><br>**Authors with competing interests**<br><br>Enter competing interest details beginning with this statement:<br><br>*I have read the journal's policy and the authors of this manuscript have the following competing interests: [insert competing interests here]*<br><br>* typeset	
**Data Availability**<br><br>Authors are required to make all data underlying the findings described fully available, without restriction, and from the time of publication. PLOS allows rare exceptions to address legal and ethical concerns. See the PLOS Data Policy and FAQ for detailed information.<br><br>A Data Availability Statement describing where the data can be found is required at submission. Your answers to this question constitute the Data Availability Statement and **will be published in the article**, if accepted.<br><br>**Important:** Stating 'data available on request from the author' is not sufficient. If your data are only available upon request, select 'No' for the first question and explain your exceptional situation in the text box.<br><br>Do the authors confirm that all data underlying the findings described in their manuscript are fully available without restriction?	Yes - all data are fully available without restriction
**Describe where the data may be found in full sentences. If you are copying our sample text, replace any instances of XXX with the appropriate details.**	All relevant data are within the manuscript and its Supporting Information files.

• If the data are **held or will be held in a public repository**, include URLs, accession numbers or DOIs. If this information will only be available after acceptance, indicate this by ticking the box below. For example: *All XXX files are available from the XXX database (accession number(s) XXX, XXX.).*<br>• If the data are all contained **within the manuscript and/or Supporting Information files**, enter the following: *All relevant data are within the manuscript and its Supporting Information files.*<br>• If neither of these applies but you are able to provide **details of access elsewhere**, with or without limitations, please do so. For example:<br><br>*Data cannot be shared publicly because of [XXX]. Data are available from the XXX Institutional Data Access / Ethics Committee (contact via XXX) for researchers who meet the criteria for access to confidential data.*<br><br>*The data underlying the results presented in the study are available from (include the name of the third party and contact information or URL).*<br>• This text is appropriate if the data are owned by a third party and authors do not have permission to share the data.<br><br>* typeset	
Additional data availability information:	

Click here to download Manuscript Manuscript.pdf

Computational models to improve surveillance for cassava brown streak disease and minimize yield loss.

Alex C. Ferris[1*], Richard O. J. H. Stutt[2], David Godding[2,3], Christopher A. Gilligan[2]

[1] Department of Bioengineering, Stanford University, Stanford, California, United States of America

[2] Department of Plant Sciences, University of Cambridge, Cambridge, United Kingdom

[3] Farming Data, Cambridge, UK

* Corresponding author

E-mail: acferris@stanford.edu (ACF)

Abstract

Cassava brown streak disease (CBSD) is a rapidly spreading viral disease that affects a major food security crop in sub-Saharan Africa. Currently, there are several proposed management interventions to minimize loss in infected fields. Field-scale data comparing the effectiveness of these interventions individually and in combination are limited and expensive to collect. Using a stochastic epidemiological model for the spread and management of CBSD in individual fields, we simulate the effectiveness of a range of management interventions. Specifically we compare the removal of diseased plants by roguing, preferential selection of planting material, deployment of virus-free 'clean seed' and pesticide on crop yield and disease status of individual fields with varying levels of whitefly density crops under low and high disease pressure. We examine management interventions for sustainable production of planting material in clean seed systems and how to improve survey protocols to identify the presence of CBSD in a field or quantify the within-field prevalence of CBSD. We also propose guidelines for practical, actionable recommendations for the deployment of management strategies in regions of sub-Saharan Africa under different disease and whitefly pressure.

Author summary

Cassava is the second largest source of calories in sub-Saharan Africa and is particularly important for the poorest farmers in the region. Cassava brown streak disease is a viral disease that causes cassava tubers to rot, rendering the roots inedible. Recently, the disease has begun to spread towards major cassava growing regions in West Africa from East Africa, where it continues to cause significant yield losses. Improved approaches for disease control are needed to enable small-holder farmers to prepare for and minimize the impact of the disease when their fields become infected. Using a combination of computational methods and mathematical models enables us to screen a much larger range of potential treatments for their likely effectiveness in managing disease and reducing crop loss than would be possible in conventional field trials, which are expensive and logistically difficult to conduct. Our results indicate that regularly planting part of the field with virus-free cassava greatly improves the yield. Removing visibly infected plants and replanting using visibly uninfected plants also improves yield, even when some of these plants may be infected but not yet showing symptoms. We also show how the survey protocol can be optimized to improve estimates of disease severity leading to more effective tailored advice to farmers in regions with different disease pressures.

Introduction

Cassava is an important food security crop in sub-Saharan Africa. Cassava is typically grown by the poorest households for reliable, subsistence calorific needs, forming the second largest source of calories overall, after maize, in sub-Saharan Africa [1]. Cassava production is currently threatened by cassava brown streak disease (CBSD), which can cause up to a 70% reduction in root yield [2]. The disease is caused by two closely related viruses, CBSV and Ugandan CBSV that can be spread by whitefly or by trading or sharing infected cuttings [3]. The disease causes subtle foliar symptoms and brown streaks on the stem as well as root necrosis [4]. Prior to 2004, CBSD was endemic to coastal Eastern Africa and Malawi; however, since 2004 CBSD has undergone a significant range expansion, spreading through Tanzania, Kenya, Uganda, Rwanda Burundi and Zambia into Central Africa, and threatening many new groups of farmers in West Africa [5].

A variety of management strategies have been proposed to limit the impact of CBSD in endemic regions and to reduce the risk of spread into new areas. Roguing plants with foliar symptoms reduces the level of within-field infection although the efficacy of roguing is highly dependent on the ability of farmers to identify disease symptoms, which, in turn, depends on the specific cassava cultivar and levels of farmer training [6,7]. Introducing virus-free planting material (known as 'clean seed') or tolerant varieties can also be used to reduce disease levels. These require less farmer education but more national-level infrastructure to produce the clean planting material and to coordinate deployment [7,8]. Preferential selection of asymptomatic cuttings is a farmer-level intervention that can potentially reduce the amount of carry-over of infection between successive crops [8]. Alternatively, spraying pesticides or soaking cuttings in pesticide can be used to lower the abundance of the whitefly vector, in order to reduce the amount of within-field spread of the virus.

Although various management techniques have been studied separately, the lack of experimental comparisons within the same study makes it difficult to know when to use one approach over another. Additionally, the effectiveness of these interventions can be highly variable when cultivars and local disease pressures differ. Measuring all of the combinations of management techniques, cultivars, and weather variables is impractical. Epidemiological models provide an alternative tool, allowing us to leverage limited amounts of experimental data in order to make inferences about the effectiveness of different interventions.

Models also help to inform the design and implementation of disease surveillance programmes. Surveillance is an important tool in improving the effectiveness of CBSD control measures, as accurate data on the location of the disease front allows more informed deployment of control measures. Several countries in Eastern Africa have conducted annual CBSD surveys by identifying plants with CBSD foliar symptoms [9]. Because of the lack of access to molecular diagnostic tools in the field and because foliar symptoms can be subtle and vary by cultivar, assessing the accuracy of field surveys remains a major challenge in monitoring disease levels of CBSD [10]. The problem is further compounded because root symptoms, which allow assessments of yield loss, only appear late in the growing season, when it is no longer possible to identify foliar symptoms. Root surveys are relatively infrequent because of greater time and cost requirements [5]. In this paper we simulate different surveillance practices within- and between-fields in order to identify how changes to current survey protocols could be used to increase accuracy. We also estimate the extent to which current protocols may underestimate the prevalence of CBSD.

We introduce and parameterize a field-level compartmental epidemiological model for the spread of CBSD with explicit vector dynamics. The model is designed to compare the spread of CBSD under conditions of different whitefly densities, local disease pressures, and when different combinations of management tools are used. The model uses experimental time-course data for the spread of the virus and whitefly dynamics [11]. Approximate Bayesian Computation (ABC) is used to estimate the unknown parameters [12]. We apply the model to simulate two agricultural situations: the first applies to individual farmer's fields, where cassava is harvested and replanted yearly and the key output is the total root yield; the second involves clean seed multiplication sites, where cassava plants are ratooned to stimulate the production of secondary stakes. The objective is to maximize the yield of clean (virus-free) stakes, while staying below a given threshold for the proportion of infected stakes. We also use the model to compare different surveillance practices by simulating visual detection of disease under different disease pressures and compare the survey results with the underlying infection state of the field.

Specifically, we address the following questions, focusing on what is realistic and practical for a given stakeholder: for a field in a region with a given vector abundance and disease pressure, what combination and intensity of management strategies are most likely to be effective in reducing the amount and yield loss from CBSD? For individual farmer fields, the effectiveness of management intervention is quantified using yield loss, and for clean seed producers, it is quantified based on the amount of clean planting material produced. For a recently infected field, such as on the epidemic front, we ask how could surveillance be optimised to improve the probability of detecting infection, taking into account the effect of vector abundance and survey variables such as the number of plants surveyed and the accuracy of the diagnostic method (e.g. visual or molecular).

Results

Individual Farmer's fields

Details of the model structure and simulation are given in the methods. The key parameter values are summarised in Table 1; treatments for managing the disease and vector are summarised in Table 2 and conditions for initial disease levels and whitefly density in Table 3.

Simulations of epidemics within individual farmer's fields were run with annual replanting over a 10 year period starting from an initial condition of either low (one infected plant per field) or high disease pressure (25% of plants infected per field) and a range of vector densities (1-20 whitefly initially evenly distributed per top five leaves of each plant). One thousand replicate simulations were run for each management intervention with random starting locations of infected plants within each replicate field. Four different types of management interventions were simulated both individually and in selected combination: clean seed, pesticide coating, roguing, and preferential selection.

Clean seed is implemented by periodically planting a portion of the field with virus-free material with an optional pesticide coating that kills whitefly at the beginning of the season. We compare two levels of clean seed implementation: lower and higher intensity implementation in which, respectively, 10% of the target field is replanted with clean seed every two years or 25% is replanted every three years (Table 2). When roguing is used for disease management, infected plants are removed during two simulated surveys per year. Preferential selection of planting material occurs midway through each season and plants without detected infection are selected to use for replanting the following year. Two efficiencies for detection of infected plants, 33% (low intensity) and 100% (high intensity), are compared for both roguing and preferential selection (Table 2).

Three combinations of interventions were simulated: all four interventions (clean seed, pesticide application, roguing and preferential selection) together as a best case scenario, the pair of behavioral interventions (roguing and preferential selection), which could be implemented by an individual farmer with the help of an extension agent, and the pair of infrastructural interventions (clean seed and pesticide coating), which could be implemented if a clean seed system were established. The yield was calculated assuming that all of the plants infected with CBSD at the end of the season have 30% of the yield compared with healthy plants [10]; the yields from replicate simulations were averaged. Selected key results from the full analysis are presented here with additional results for reference in the Supplementary Information.

When simulating roguing, there are two distinct outcomes in individual simulations:

either all of the infected plants are rogued and the field is free of CBSD, or the field becomes fully infected and there is a negative effect on yield compared with doing nothing because of additional loss of yield from the removal of plants by roguing. Even with perfect roguing accuracy and starting with a single infected plant, fields becomes fully infected in approximately 75% of the simulations because the incubation period results in a large number of infected plants that have yet to develop visible symptoms early enough during the roguing period (Fig 1b). High accuracy roguing, by which we mean infected plants are always detected if surveyed, is more likely to eliminate CBSD from the field but when elimination fails, continued roguing would eventually lead to the nonsensical result of removing all plants leading to complete loss of yield (Fig 1b,d). Overall, roguing only increases the average yield when there is very low whitefly abundance and there is either low disease pressure or high disease pressure with high accuracy roguing (Fig S1).

Fig 1. Effect of high and low roguing accuracy in detecting and removing infected plants on potential cassava yield loss across multiple years. One thousand independent simulation runs are shown for each treatment combination of roguing accuracy x initial CBSD density and initial density of five whitefly per top five leaves per plant. (A,B) the initial CBSD infection is a single plant; (C,D) initial CBSD infection is 25% of plants per field. (A,C) have a low roguing accuracy; (B,D) have high roguing accuracy (see Table 2). Darker intensity lines indicate multiple simulation lines overlapping. For each sub-plot, the x-axis is the time since the epidemic began, with each season demarcated by vertical dashed lines: the y-axis is the potential yield during that season relative to a healthy field if no additional plants are infected or rogued (see text for details). The final effective yield for a season is given by the value of yield at the end of a season.

Our results indicate that the deployment of clean seed was more successful in reducing potential crop loss from CBSD than other separately applied management interventions (roguing of symptomatic plants and preferential selection of planting material) (Fig S1). The yield advantage of using clean seed decreased with increasing vector densities, necessitating the introduction of more clean seed planting material for effective control even at five whitefly on upper leaves per plant (Fig 2b,c). The effect of clean seed on yield is also much larger when planted as a single block in the corner of a field as opposed to being randomly distributed throughout the field (cf Fig 2 for blocked and Fig S2 for random introduction of clean seed). In the simulations, randomly distributed cuttings are quickly infected by neighboring infected plants, whereas it takes longer for the infection to spread completely through a large block of uninfected clean seed.

Fig 2. The average yield of a field with different management interventions. For each plot, the x-axis is the number of seasons since the start of the epidemic, and the y-axis is the average yield over an ensemble of 1000 epidemics for each management intervention. (A,B,C,D,E) have a starting infection of a single plant while (F,G,H,I,J) start with 25% of the field infected. (A,F) have no interventions, (B,G) use low intensity clean seed, (C,H) use high intensity clean seed, (D,I) use all interventions at low intensity, and (E,J) use all interventions at high intensity (see Table 2).

Almost all the management interventions increased yield to some extent but only at the

lowest vector density; the exception being low intensity roguing in endemic fields (cf Fig S1). Preferential selection of planting material on its own had a minimal impact on yield, and in combination with roguing decreased yield except for the lowest vector density (Fig S1). When combining the high intensity interventions, the fields were rendered free of CBSD within three years with low starting CBSD infection (Fig 2e).

Regional management recommendations

We propose the following field level recommendations for management interventions to use in different regions of sub-Saharan Africa based on the likely disease pressure, crop density, and whitefly abundance. Exploratory simulations showed that epidemic predictions behaved differently at low and high disease pressures according to whitefly abundance. Survey data were used to classify locations with respect to (very low to high) whitefly abundance [13,14]. High disease pressure areas were defined as locations within 500km radius of a known CBSD positive survey point or in regions with high cassava density [5,15–23]. Therefore, parts of Nigeria have been classified as having high disease pressure, even though CBSD has not been reported in West Africa, based on the assumption that if CBSD reaches the region the disease would spread more quickly in places with high cassava density. Other regions were classified as having low disease pressure.

In most of West Africa there is low whitefly abundance, which increases the effectiveness of all the management interventions, particularly roguing and preferential selection (Fig 3 and Fig S1). In regions with moderate whitefly but low disease pressure, roguing and preferential selection become less effective, but clean seed and pesticide coating are still effective at both low and high intensities (Fig 3 S1). With higher disease pressure and whitefly abundance, high intensity clean seed and pesticide coating are needed to improve yield (Fig 3 and data in S1 Figure).

Fig 3. Recommended interventions for improving yield in CBSD infected fields in selected regions based on whitefly abundance, host density, and disease pressure. Asterisks indicate regions that do not currently have CBSD and what the disease pressure is likely to be if CBSD were present. Recommendations are listed in order of decreasing impact on yield. For locations A and B, low intensity interventions are effective but high intensity would work better; however, for location C only high intensity is likely to be effective (see Table 2).

Multiplication in clean seed nurseries

Given evidence for the effectiveness of clean seed as a management intervention, we also simulated how to use management interventions effectively to increase the output and sustainability of clean seed multiplication. Unlike within farmer's fields, in these simulations, nursery fields for the production of clean planting material are ratooned every three years. The specific simulation settings were based on the local level of clean seed multiplication for nurseries from Tanzania's proposed clean seed system for which fields are used for supply of planting material up to a threshold level of 10% within-field infected plants [24] (L. Good, pers. com.).

Roguing and pesticide coating on newly planted clean seed each increased the number of clean cuttings generated compared with no interventions under low whitefly abundance (Fig 4).

With high whitefly abundance, however, using pesticide resulted in a small improvement, and there is no improvement with low accuracy roguing. Increasing the roguing accuracy from low to high also led to a large improvement in the average number of cuttings generated, suggesting that cassava varieties where it is easier to detect foliar symptoms should be prioritized for use in clean seed systems to improve sustainability over time (Fig 4b,f). There was also a strong synergistic effect when combining roguing and pesticide treatment for all levels of whitefly abundance (Fig 4).

Fig 4. Average number of cuttings produced by fields for clean seed production with different management interventions. For each plot, the x-axis is the number of seasons, and the y-axis is the average number of cuttings produced in a field over an ensemble of 1000 simulations. (A,E) are the no intervention baseline results, (B,C,D) have a low intervention intensity, and (F,G,H) have a high intervention intensity (see Table 2). All simulations relate to low initial density of one infected plant per field.

Surveillance

We used the simulation model to compare the effectiveness of a range of within-field surveillance practices in successfully detecting CBSD disease in infected fields and in estimating the level of within-field disease incidence. The basic survey technique is described in the methods section and follows the protocol described by Sseruwagi *et al.* [9]. We simulated the effects of different combinations of sampling intensity (numbers of plants assessed for disease) and accuracy (correctly assigning an infected plant as diseased) on the probability of detecting disease.   In practice survey accuracy can vary depending on the type of diagnostic test used to assess disease (e.g. root symptoms, foliar symptoms, or molecular diagnostics). Accuracy also depends on the severity of foliar symptoms in the local cultivars, and on surveyor expertise.

For a starting state of 5% of plants infected however, surveying at least 30 plants per field and conducting foliar surveys later instead of at the beginning of the growing season results in a high probability of detecting CBSD in a field regardless of survey accuracy or whitefly abundance (Fig 5). Conducting surveys later in the season greatly improves the probability of detecting infection in all cases except with a very low whitefly density (one whitefly per plant) because CBSD within-field abundance increases enough over the course of the growing season to improve the probability of detecting the infection (Fig 5). The probability of detecting CBSD is very low when there is only one infected cutting in the field regardless of survey accuracy, survey timing, or the number of plants surveyed (Fig S1). Similarly, when greater than 20% of the field are infected, the survey will almost always detect CBSD irrespective of the survey parameters (results not shown).

Fig 5. The probability of detecting infection in a field with an initial within-field incidence of 5%. For each plot, the x-axis is the average number of whitefly per top five leaves on the plant, and the y-axis is a different combination of survey variables (surveyor accuracy and number of plants surveyed). Simulated surveys were conducted at (A) 90 days after planting; (B) 180 days after planting; (C) 299 days after planting.

Estimating the within-field prevalence of CBSD in fields where the disease is established

is highly dependent on survey accuracy, but not on the number of plants surveyed in the field (Fig 6). Conducting foliar surveying later in the season has a moderate effect on the proportional error of the estimates for simulated epidemics, but only for fields infected with low levels of CBSD (Fig 6). This implies that replacing standard foliar surveys with molecular diagnostics or root surveys at the end of the season (each of which is associated with greater accuracy)could increase survey accuracy, but in general, estimating the prevalence of CBSD in fields with an initial infection of less than 20% will have a high proportional error.

Fig 6. The percent error of within-field CBSD prevalence estimated from a survey compared with the true prevalence at the end of the season. For each plot, the x-axis is the average number of whitefly per top five leaves on the plant, and the y-axis is a different combination of survey variables (surveyor accuracy number of plants surveyed). (A,D,G) have surveys conducted 90 days after planting, (B,E,H) have surveys conducted 180 days after planting, and (C,F,I) have surveys conducted 299 days after planting. (A,B,C) have a starting infection of 5%, (D,E,F) have a starting infection of 20% and (G,H,I) have a starting infection of 50%.

Discussion

Our study demonstrates that roguing, preferential selection of asymptomatic planting material, and clean seed are all individually effective at reducing epidemic spread and improving yield in fields within areas of low disease pressure and low whitefly abundance. In regions with high whitefly abundance or high disease pressure, the use of clean seed is essential to improve average yield. Clean seed nurseries were able to remain below a critical CBSD infection limit of 10% for several periods in regions with low to moderate whitefly abundance, particularly when roguing or pesticide coating are also used. Our results also indicate that surveillance to detect CBSD when it is present at low abundance (5%) can be optimized by conducting surveys later in the growing season and surveying at least 30 plants per field.

Individual Farmer's fields & clean seed multiplication

We simulated two different types of management interventions, those that depend on farmer education (roguing and preferential selection), and those that rely on external inputs (clean seed and pesticide coating). Current guidelines for cassava recommend selecting healthy plants mid-season and roguing diseased plants [25,26]. However, previous studies show that farmers in regions with CBSD are unaware of the disease and that training from extension agents is required for farmers to be able to recognize infected plants in their fields [8,27]. Based on our simulation results, preferential selection of asymptomatic planting material does not decrease the average yields, but only results in a large yield increase in regions that have very low levels of whitefly and for plants with easily detectable foliar symptoms (Fig 3, S1). Assuming that the practice increases accuracy of detection, training farmers to select plants at the end of the season based on root symptoms as opposed to selecting plants midseason using foliar symptoms [25] would be expected to increase the number of regions that can benefit from preferential selection.

The effectiveness of roguing has been tested previously in field trials in conjunction with planting clean seed: the practice was beneficial in one trial location and had no effect on yield in the other location [7]. Our simulation data suggest that when there is moderate or high whitefly abundance, roguing decreases yield, even with perfect accuracy (Fig S1). Instead of only roguing

infected plants, shifting to a strategy of also removing plants within a set radius [28] of any plants that are found to be symptomatic may increase the efficacy when there is higher whitefly abundance. The optimal radius for roguing [cf 28] would likely be highly dependent on disease pressure and whitefly population levels, and in the interest of brevity this has been left for future work.

The simulations suggest that using high intensity clean seed (replanting a quarter of the field with clean seed every other year) always improves average yield, and that in regions with very low abundance of whitefly, less frequent input is also beneficial (Fig 2). Legg *et al.* [7] also reported yield improvements in field trials from introducing clean seed in a variety of locations with different whitefly abundance and disease pressure. The simulation results also suggest that sustainably multiplying uninfected cuttings to use as input clean seed for farmers is feasible using the proposed Tanzanian clean seed system (replanting a third of the field every year with a within-field infection limit of 10%), as long as the multiplication is done in areas with low whitefly and roguing or pesticide is used (Fig 4) [24] (L. Good, pers. com.). These conclusions support previous simulation results by McQuaid *et al.* [6] that clean seed multiplication is sustainable in regions with low disease pressure and whitefly density, particularly when there is effective roguing. However, it is difficult to make more detailed comparisons because McQuaid *et al.* [6] used a very different set of parameters based on cassava mosaic disease and assumed that roguing occurred at least monthly with at least 50% accuracy.

Planting of clean seed can also improve yield by distributing cultivars that have other beneficial characteristics. Although truly resistant CBSD varieties do not currently exist, tolerant cultivars can be used to minimize the yield loss in infected plants [29,30]. Other important traits include clear foliar symptoms to increase the effectiveness in detecting infection and hence the impact of roguing and preferential selection. Cultivars that support lower levels of whitefly to reduce vector driven spread of CBSD, and cultivars with early bulking roots to reduce the length of each season and minimize the amount of root necrosis [8,31] are also likely to be effective in mitigating disease and crop loss.

Surveillance

The model results suggest that when seeking to detect the presence or absence of CBSD in a field, the survey protocol is particularly important at relatively low within-field CBSD abundance. With higher vector abundance, there is an advantage to surveying later in the season because there is enough within-field CBSD spread during the season to meaningfully increase the probability of detecting an infected plant (Fig 5). Surveying at least 30 plants per field with a survey accuracy of at least 50% is important, but further increasing the survey accuracy has a minimal impact (Fig 5). Using molecular diagnostics in conjunction with visual symptom observation or conducting surveys using root symptoms instead of foliar symptoms (to improve accuracy of detection) could be a way to extend the window of time for doing surveys that have a high probability of detecting an infection. In practice, most fields are probably not identified by surveys until closer to 5% of the plants are infected (Fig S1). This threshold for detecting infected fields suggests that there is a lag between the disease front and detection of infected fields, and that control efforts should be expanded beyond the region where infected plants have been detected.

Accurately estimating the within-field prevalence of CBSD with surveys is a more difficult problem. When the within-field prevalence is 5% or less, the percent error is high regardless of the survey variables used. Even for fields with a starting infection of 20% or 50%,

the vector prevalence, survey timing, the number of plants surveyed all have a small effect on percent error. Survey accuracy is by far the most important of the variables under surveillance control, suggesting that a survey protocol that analyzes ten plants using molecular diagnostics would be a reasonably effective approach (Fig 6). Due to the large differences in optimal survey protocols for estimating whether a field is infected and for estimating within field prevalence, it is not feasible to meet both objectives using a single survey data protocol.

In conclusion, we find that for much of East and West Africa, preferential selection, roguing, and clean seed can all improve yield, while clean seed is required to improve yield in areas with high disease pressure or whitefly abundance. Our findings also demonstrate that clean seed can be generated sustainably in moderate to low whitefly areas, particularly when roguing or pesticide coating is used. Surveys to detect the presence of CBSD can be highly accurate even close to the epidemic front, allowing for effective targeting of management interventions. However, accurately quantifying the within-field prevalence requires a higher survey accuracy than is unlikely to be achieved using foliar symptoms alone.

Methods

Modelling approach

Plants become infected in a field either through viruliferous whitefly or planting already infected cuttings. In the model, plants are initially cryptically infected with a 30 day lag before symptoms start to become visible at which point symptoms increase linearly until 90 days after infection. This reflects results from Mware et al. [15] that foliar symptoms from cassava brown streak virus develop 26-60 days after exposure to infected whitefly (Table 1, Fig 7A). We also assume that when a plant initially becomes infected there is zero chance for emigrating whitefly to carry the virus, and that the infectiousness of a plant increases linearly until it becomes maximally infectious after 90 days, reflecting the experimental results of Rwegasira and Rey [32] (Table 1, Fig. 7A). Field dimensions and other critical parameters are summarized in Table 1.

We model the spread of CBSD within a field using a spatially-explicit, individual-based stochastic model that explicitly models individual cassava plants and whitefly in a field. Each plant holds a population of whitefly. Whitefly movements between plants are updated in daily time steps, and each whitefly has a chance to fly to another plant every day (Fig. 7B). The destination plant for a whitefly movement is chosen by a weighted random sample with each potential destination weighted by an exponential kernel. As whitefly only transiently carry the CBSV virus, a check is made upon whitefly emigration from an infectious plant to see whether the insect is carrying the virus. Reflecting results from Maruthi et al. [14] showing that with intermittent feeding CBSV is retained for less than 24hrs, we assume that whitefly only retain the virus for the first feeding after dispersal because in the model whitefly only move between plants once a day.
When parameterizing the model, the maximum transmission probability was set at 12.5% reflecting experimental results [15,33,34].

At the beginning of a growing season, replanting of a field is simulated by selecting cuttings from the previous year, and the initial level of within-field infection is

the average of the infection of the selected plants and any clean seed (Fig 7B). On each day during the growing season, whitefly move between plants, new plants become infected by whitefly, and the symptom severity and infectiousness increase. Roguing infected plants and preferential selection of plants to use in the subsequent growing season can also occur midway through the growing season (Fig 7B). At the end of the season, the yield of the field is calculated using a simple relationship between infection and yield assuming that a CBSD infected plant has 30% of the yield compared with a healthy plant [10] (Fig 7B).

Fig 7. Overview of model structure. (A) shows the infection process for hosts progressing from susceptible and uninfected to cryptically infected. (B) shows the model over the course of a single growing season. At the start of the simulation, the field is initialized with the location of infected plants and whitefly. Roguing and preferential selection occur at set intervals during the season whereas whitefly movement, new infections, and intra-plant symptom and infection increases occur daily.

Table 1. Parameter values that were constant in all simulations.

Parameters	Parameter value
Growing season length	300 days
Plants in field	120 x 50
Spacing within rows	1m
Spacing between rows	1.5m
Plants fully infected	90 days after infection
Symptom delay after infection	30 days after infection
Plants fully symptomatic	90 days after infection
Number of cuttings per plant	10

Table 2. Description of management interventions[1] at low and high intensity.

Intervention	Description	Low intensity	High intensity
Roguing	Removing symptomatic plants at 3 and 6 mo after planting	33% accuracy in detecting symptoms of infection	100% accuracy in detecting symptoms of infection
Preferential selection	Selection of asymptomatic stems at 180 days after planting for subsequent planting	33% accuracy in detecting symptoms of infection	100% accuracy in detecting symptoms of infection

Clean seed	Planting part of the field with virus free plants	10% of field every three years (L. Good, pers. com.)	25% of field every two years (L. Good, pers. com.)
Pesticide coated clean seed	Planting clean seed coated with pesticide	Lasts for 42 days after planting (J. Colvin, pers. com.)	Lasts for 56 days after planting (J. Colvin, pers. com.)

[1]Resource requirements are indicated by shading where light grey indicates farmer education and dark grey indicates infrastructure (commercial seed system, pesticide availability).

Table 3. Summary of disease pressure and whitefly settings for simulations with low or high disease pressure.

**Epidemiological driver**	**Value**
Low disease pressure	1 infected plant per field
High disease pressure	25% infected plants per field
Whitefly	{1, 5, 10, 15, 20} whitefly per top 5 leaves of plant

Simulating clean seed nurseries

When simulating clean seed nurseries, ratooning is used instead of replanting at the end of the growing season. To simulate ratooning, the field is divided into sections, which are replanted every three years using clean seed, and any plants removed by roguing are not replaced until the section of the field is replanted. This protocol mimics the guidelines given to cassava seed entrepreneurs in the clean seed system being developed by Tanzanian scientists in partnership with the Mennonite Economic Development Associates [24]. Fields are removed entirely when more than 10% of the plants are infected at the end of season (L. Good, pers. comm.). The effectiveness of different management techniques was quantified by calculating the number of cuttings produced by a field. All live plants produced ten cuttings and rogued plants produced zero cuttings (see Table 1). If the average CBSD infection in the cuttings was above the cutoff value of 10%, the field produced no cuttings for the rest of the simulation (L. Good, pers. com.).

Modelling management interventions

Roguing is modelled by simulating a survey of all plants in a field, then removing any plants detected as symptomatic three and six months into the growing season (Table 2). For preferential selection, plants are surveyed 180 days after planting, and a subset of plants without visible symptoms is pre-selected as planting material for the following year (Table 2). A scale factor can be applied to the plant symptom value to mimic imperfect surveillance or cultivars with less obvious foliar symptoms.

The model allows for flexibility in selecting planting material: for example, clean seed (i.e. uninfected plants) can be added to specific regions of a field, and preferential selection starts

by trying to choose plants from the clean seed region of the field. Clean seed can be combined with a pesticide coating, which is modelled by killing any whitefly that land on the plant before the pesticide loses effectiveness midway through the season (J. Colvin, pers. com.). To maintain a constant number of whitefly per field, a new whitefly is added to a random plant after one is killed by pesticide and more whitefly are added to the plants when the pesticide efficacy declines to bring the plants up to the field average. The differences between the low and high intensity versions of all four interventions are summarised in Table 2, and the differences in initial conditions for the simulations are recorded in Table 3. The efficacy of the different management techniques were compared by estimating the average yield of a field over time, using a simple relationship between infection and yield assuming that a CBSD infected plant has 30% of the yield compared with a healthy plant [10].

Modelling surveys

To simulate surveys, we assume that the probability of detecting an infected plant is proportional to the extent of symptoms for that plant. Surveys select only a subset of N randomly selected plants approximately uniformly spaced along two diagonal transects of a field [9].

Data

Data for disease progression time courses and monthly whitefly counts come from Katono *et al.* [11]. The data were collected at two different locations in Uganda, Wakiso (high CBSD prevalence) and Kamuli (medium CBSD prevalence). At each test site, the CBSD susceptible TME204 cassava variety was grown in 100 plant blocks in a 10 x10 square lattice with 1m between rows of plants. There were four replicate blocks with 2m between each block. Researchers visually surveyed the plants for foliar symptoms and counted the number of whitefly monthly from 1 to 12 months after planting.

Parameter estimation

Approximate Bayesian computation (ABC) methods [12] were used to estimate the epidemiological parameters from the Kamuli dataset [11]. Whitefly abundance was assumed to be the average of the recorded values with no seasonal dynamics. Three parameters were estimated using the Kamuli dataset [11]: (i) probability of a whitefly leaving a plant per day; (ii) probability of a whitefly infecting a new plant; (iii) dispersal kernel scale factor for an exponential kernel. We used the summary statistic: $\sum \frac{(experimental\ value - simulated\ value)^2}{experimental\ value}$ (analogous to the $\chi 2$ statistic) and explored a range of tolerance values, selecting 15 as a robust measure (S5).

To validate that the posterior parameter values were robust to different field conditions, we used the parameters obtained from fitting to the Kamuli dataset and ran simulations using the field conditions from the training dataset, Kamuli, and the validation dataset, Wakiso. The percentage of simulation runs with a summary statistic value below the cutoff were higher for the validation dataset than the training dataset, suggesting that the parameters generalize beyond a single geographic location (Fig S6).

Acknowledgements

The authors gratefully acknowledge discussion with Professor John Colvin on whitefly behaviour and with Dr Lauren Good on strategies for clean seed deployment. This work was supported by a grant to CAG, an internship to AF from the Bill and Melinda Gates Foundation, and a fellowship to AF by the Fannie and John Hertz Foundation.

References

1. FAO, IFAD. A review of cassava in Africa with country case studies on Nigeria, Ghana, the United Republic of Tanzania, Uganda and Benin. Rome, Italy: International Fund for Agricultural Development Food and Agriculture Organization of the United Nations; 2005.

2. Hillocks RJ, Raya MD, Mtunda K, Kiozia H. Effects of brown streak virus disease on yield and quality of cassava in Tanzania. J Phytopathol. 2001;149: 389–394. doi:10.1046/j.1439-0434.2001.00641.x

3. Winter S, Koerbler M, Stein B, Pietruszka A, Paape M, Butgereitt A. Analysis of cassava brown streak viruses reveals the presence of distinct virus species causing cassava brown streak disease in East Africa. J Gen Virol. 2010;91: 1365–1372. doi:10.1099/vir.0.014688-0

4. Nichols RFW. The brown streak disease of cassava. East Afr Agric J. 1950;15: 154–160. doi:10.1080/03670074.1950.11664727

5. Alicai T, Omongo CA, Maruthi MN, Hillocks RJ, Baguma Y, Kawuki R, et al. Re-emergence of cassava brown streak disease in Uganda. Plant Dis. 2007;91: 24–29.

6. McQuaid CF, Sseruwagi P, Pariyo A, van den Bosch F. Cassava brown streak disease and the sustainability of a clean seed system. Plant Pathol. 2016;65: 299–309. doi:10.1111/ppa.12453

7. Legg J, Ndalahwa M, Yabeja J, Ndyetabula I, Bouwmeester H, Shirima R, et al. Community phytosanitation to manage cassava brown streak disease. Virus Res. 2017. doi:10.1016/j.virusres.2017.04.020

8. Chipeta MM, Shanahan P, Melis R, Sibiya J, Benesi IRM. Farmers' knowledge of cassava brown streak disease and its management in Malawi. Int J Pest Manag. 2016;0: 1–10. doi:10.1080/09670874.2016.1167268

9. Sseruwagi P, Sserubombwe WS, Legg JP, Ndunguru J, Thresh JM. Methods of surveying the incidence and severity of cassava mosaic disease and whitefly vector populations on cassava in Africa: a review. Virus Res. 2004;100: 129–42. doi:10.1016/j.virusres.2003.12.021

10. Hillocks R, Maruthi M, Kulembeka H, Jeremiah S, Alacho F, Masinde E, et al. Disparity between leaf and root symptoms and crop losses associated with cassava brown streak disease in four countries in Eastern Africa. J Phytopathol. 2015;2004: 86–93. doi:10.1111/jph.12430

11. Katono K, Alicai T, Baguma Y, Edema R, Bua A, Omongo C. Influence of host plant resistance and disease pressure on spread of cassava brown streak disease in Uganda. Am J Exp Agric. 2015;7: 284–293. doi:10.9734/AJEA/2015/15563

12. Beaumont MA. Approximate Bayesian computation in evolution and ecology. Annu Rev Ecol Evol Syst. 2010;41: 379–406. doi:10.1146/annurev-ecolsys-102209-144621

13. Jeremiah SC. The role of the whitefly (*Bemisia tabaci Gennadius*) in the transmission and spread of cassava brown streak and cassava mosaic viruses in the field and semi field conditions. PhD Thesis, PhD Thesis, University of Dar es salaam. 2014.

14. Maruthi MN, Jeremiah SC, Mohammed IU, Legg JP. The role of the whitefly, *Bemisia tabaci (Gennadius)*, and farmer practices in the spread of cassava brown streak Ipomoviruses. J Phytopathol. 2017; 707–717. doi:10.1111/jph.12609

15. Mware B, Narla R, Amata R, Olubayo F, Songa J, Kyamanyua S, et al. Efficiency of cassava brown streak virus transmission by two whitefly species in coastal Kenya. J Gen Mol Virol. 2009;1: 40–45.

16. Maruthi M, Hillocks R. Transmission of cassava brown streak virus by *Bemisia Tabaci (Gennadius)*. J Phytopathol. 2005;312: 307–312.

17. Maruthi MN, Bouvaine S, Tufan HA, Mohammed IU, Hillocks RJ. Transcriptional response of virus-infected cassava and identification of putative sources of resistance for cassava brown streak disease. PLOS ONE. 2014;9: e96642. doi:10.1371/journal.pone.0096642

18. Pacific P. Commercially sustainable, quality assured cassava seed system in Tanzania: A pilot innovation project. Sweet Potato Seed Systems Community of Practice; 2016 May 10; Corrider Springs, Arusha Tanzania.

19. Eni AO, Efekemo OP, Soluade MG, Popoola SI, Atayero AA. Incidence of cassava mosaic disease and associated whitefly vectors in South West and North Central Nigeria: Data exploration. Data Brief. 2018;19: 370–392. doi:10.1016/j.dib.2018.05.016

20. Sseruwagi P, Tairo F, Ndunguru J. The Cassava Diagnostics Project (CDP): A review of 10 years of research 2008–2018. Dar es Salaam, Tanzania: Tanzania Agricultural Research Institute (TARI)–Mikocheni; 2018.

21. Ntawuruhunga P, Legg J. New spread of cassava brown streak virus disease and its implications for the movement of cassava germplasm in the East and Central African region. USAID Crop Crisis Control Proj C3P. 2007.

22. Mbanzibwa DR, Tian YP, Tugume a. K, Mukasa SB, Tairo F, Kyamanywa S, et al. Genetically distinct strains of cassava brown streak virus in the Lake Victoria basin and the Indian Ocean Coastal Area of East Africa. Arch Virol. 2009;154: 353–359. doi:10.1007/s00705-008-0301-9

23. Szyniszewska AM. CassavaMap, a fine-resolution representation of cassava production and harvested area in Africa in 2014. Sci Data. Submitted.

24. Bigirimana S, Barumbanze P, Ndayihanzamaso P, Shirima R, Legg JP. First report of cassava brown streak disease and associated Ugandan cassava brown streak virus in Burundi. New Dis Rep. 2011;24: 2044–0588. doi:10.1094/PD-91-0024

25. Mulimbi W, Phemba X, Assumani B. First report of Ugandan cassava brown streak virus on cassava in Democratic Republic of Congo. New Dis Rep. 2012; 5197. doi:10.5197/j.2044-0588.2012.026.011

26. Munganyinka E, Ateka EM, Kihurani AW, Kanyange MC, Tairo F, Sseruwagi P, et al. Cassava brown streak disease in Rwanda, the associated viruses and disease phenotypes. Plant Pathol. 2018;67: 377–387. doi:10.1111/ppa.12789

27. Mulenga RM, Boykin LM, Chikoti PC, Sichilima S, Ng'uni D, Alabi OJ. Cassava brown streak disease and Ugandan cassava brown streak virus reported for the first time in Zambia. Plant Dis. 2018;102: 1410–1418. doi:10.1094/PDIS-11-17-1707-RE

28. Legg J, Bouwmeester H. Cassava disease surveillance surveys 2009 mapping report Great Lakes Cassava Initiative text and mapping. Surv Team Leadersh Simon Bigirimana Burundi Hannington Obiero Kenya Gervais Gashaka Rwanda. 2010.

29. Jorge MA. Regeneration guidelines: Cassava. Dulloo ME, Thormann I, Jorge MA, Hanson J, editors. Rome, Italy: CGIAR System-wide Genetic Resource Programme, Rome; 2008.

30. Chiona M, Ntawuruhunga P, Mukuka I, Chalwe A, Phiri N, Chikote PC, et al. Growing cassava: Training manual for extension and farmers in Zambia. Zambia: International Institute of Tropical Agriculture (IITA); 2016.

31. Kumakech A. Knowledge on cassava disease management: The case of cassava brown streak disease awareness in Northern Uganda. Afr J Plant Sci. 2013;7: 597–601. doi:10.5897/AJPS2013.1098

32. Rwegasira GM, M. E. Rey C. Response of selected cassava varieties to the incidence and severity of cassava brown streak disease in Tanzania. J Agric Sci. 2012;4: 237–245. doi:10.5539/jas.v4n7p237

33. Patil BL, Delhi N, Delhi N, Patil BL, Patil BL, Legg JP, et al. Cassava brown streak disease: A threat to food security in Africa. J Gen Virol. 2014;96: 956–968. doi:10.1099/vir.0.000014

34. Kawuki RS, Kaweesi T, Esuma W, Pariyo A, Kayondo IS, Ozimati A, et al. Eleven years of breeding efforts to combat cassava brown streak disease. Breed Sci. 2016;66: 560–571. doi:10.1270/jsbbs.16005

Supplemental Figures

S1 Fig. The difference in average yield between fields with management interventions and a field with no management interventions. (A,B,C,D,E) start with one infected plant and use low intensity interventions, (F,G,H,I,J) start with one infected plant and use high intensity interventions, (K,L,M,N,O) start with a quarter of the field infected and use low intensity interventions, and (P,Q,R,S,T) start with a quarter of the field infected and use high intensity interventions. For each subplot, the x-axis is the number of seasons since the start of the epidemic.

S2 Fig. The average yield of a field using randomly planted clean seed. For each plot, the x-axis is the number of seasons since the start of the epidemic, and the y-axis is the average yield over an ensemble of 1000 epidemics for each management intervention. (A,B,C) have a starting infection of a single plant while (D,E,F) start with 25% of the field infected. (A,D) have no interventions, (B,E) use low intensity randomly planted clean seed, and (C,F) use high intensity randomly planted clean seed.

S3 Fig. The probability of detecting infection in a field with an initial within-field incidence of 1 infected plant. For each plot, the x-axis is the average number of whitefly per top five leaves on the plant, and the y-axis is a different combination of survey parameters (surveyor accuracy and number of plants surveyed). The surveys were conducted at (A) 90 days after planting; (B) 180 days after planting; (C) 299 days after planting.

S4 Fig. An example time course for a field with no management interventions and an average of 15 whitefly/top five leaves. Uninfected plants are shown in navy, infected plants are shown in yellow.

S5. Sample simulation results and posterior probabilities for the parameters used in the simulations. (A) shows experimental data from Katono *et al.* in cyan with an example of simulated data with an accepted set of parameters shown as a red line. Double headed arrows show the distance between the experimental and simulated data. (B,C,D) show marginal posterior probabilities for the parameter values.

S6 Fig. The distribution of summary statistic scores from 5000 simulation runs using the accepted parameters and whitefly abundance from (A) Kamuli or (B) Wakiso. The red line indicates a summary statistic score of 15. Thirty one percent of the Wakiso simulations are below the cutoff value of 15 and 15% of the Kamuli simulations are below the cutoff.

Click here to download Figure Fig1.tif

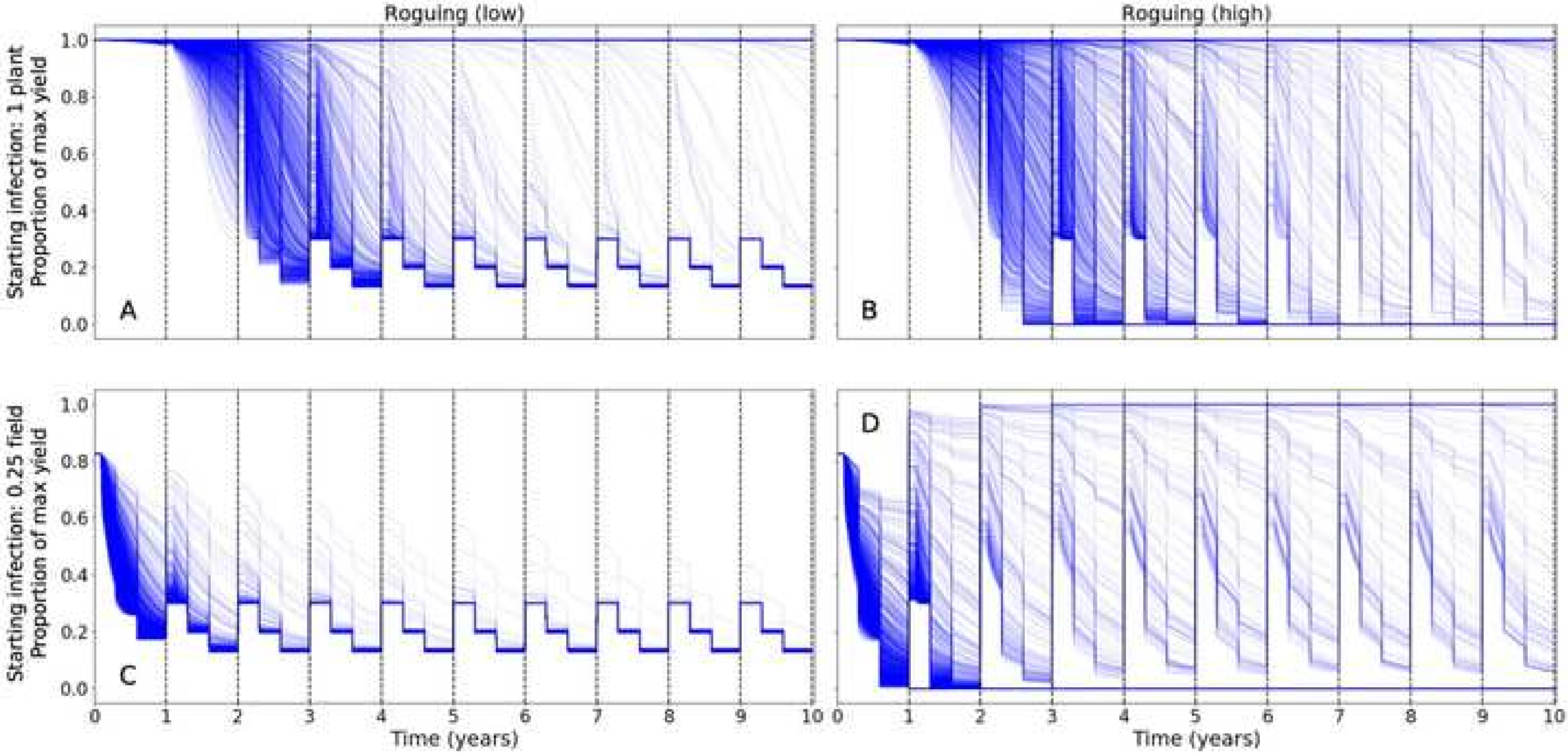

Click here to download Figure Fig2.tif

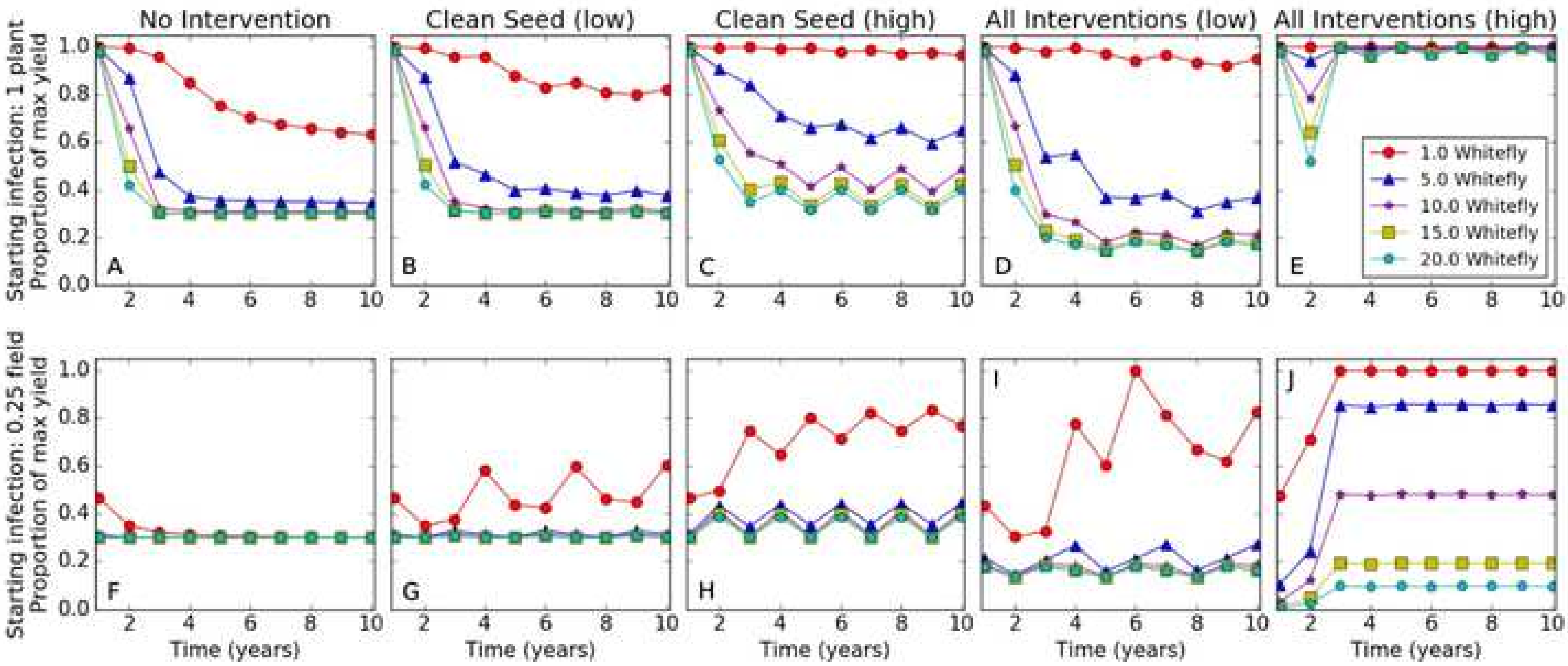

Click here to download Figure Fig3.tif

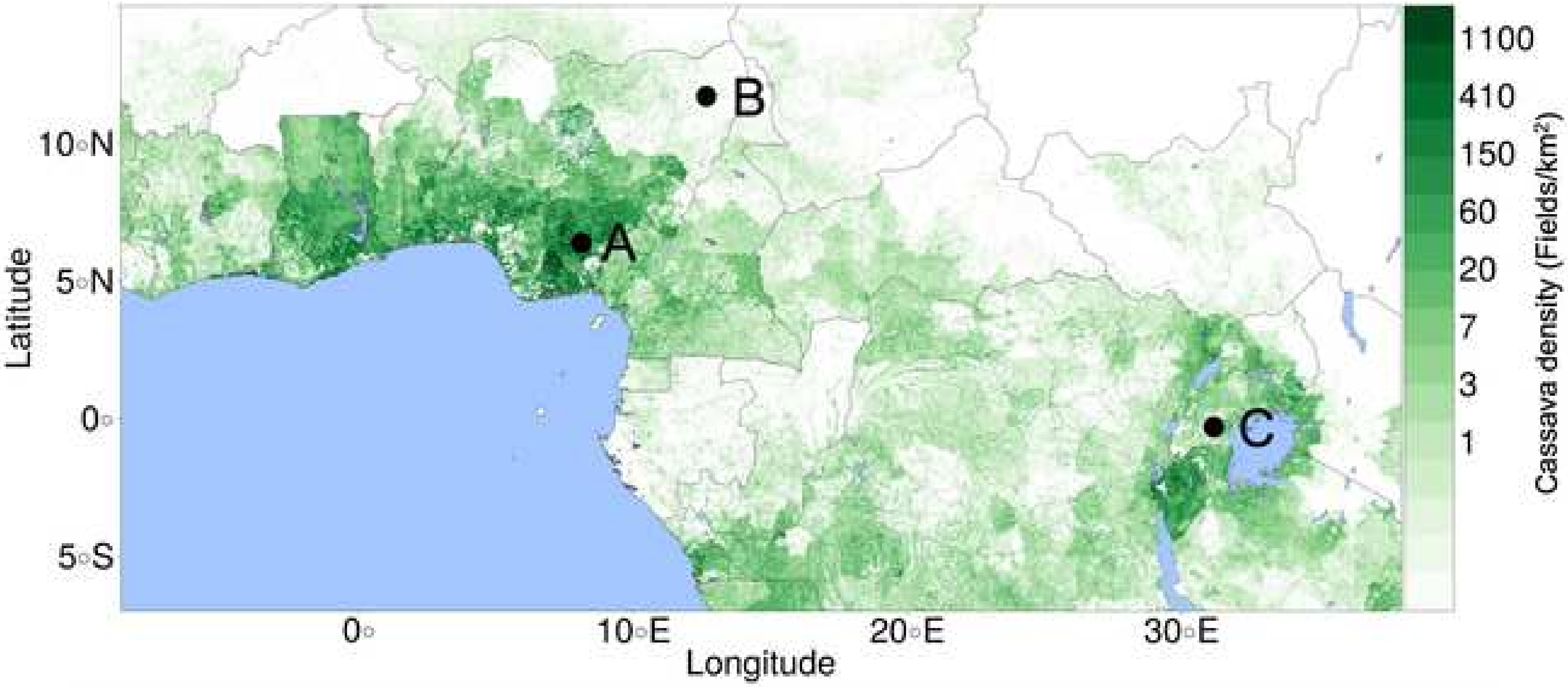

Location	Whitefly abundance	Local Disease pressure	Recommendations in Order of Effectiveness
A	Very low	High*	1. Clean seed<br>2. Clean seed with pesticide coating<br>3. Roguing<br>4. Preferential selection
B	Moderate	Low*	1. Clean seed<br>2. Clean seed with pesticide coating
C	High	High	1. High intensity clean seed<br>2. High intensity clean seed with pesticide coating

Click here to download Figure Fig4.tif

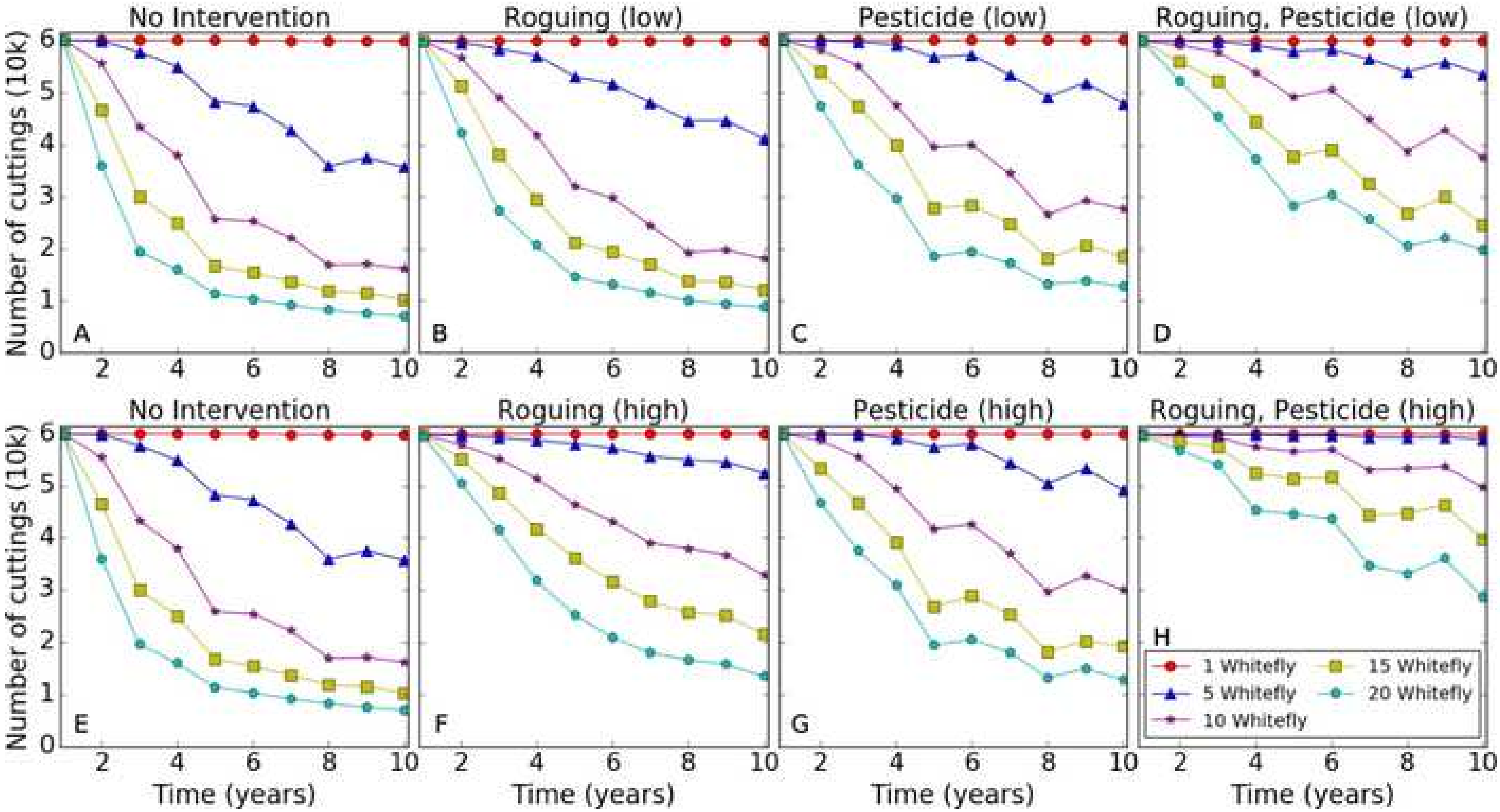

Click here to download Figure Fig5.tif

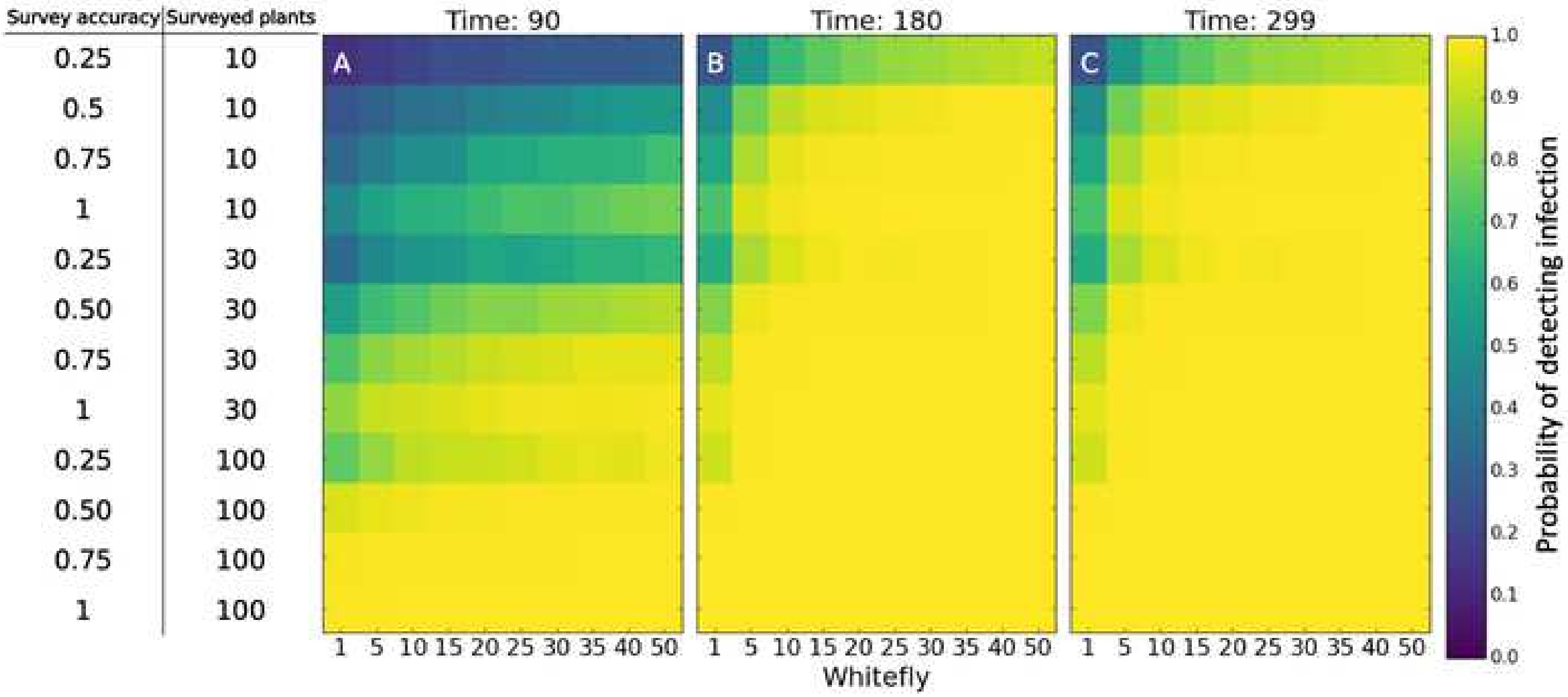

Click here to download Figure Fig6.tif

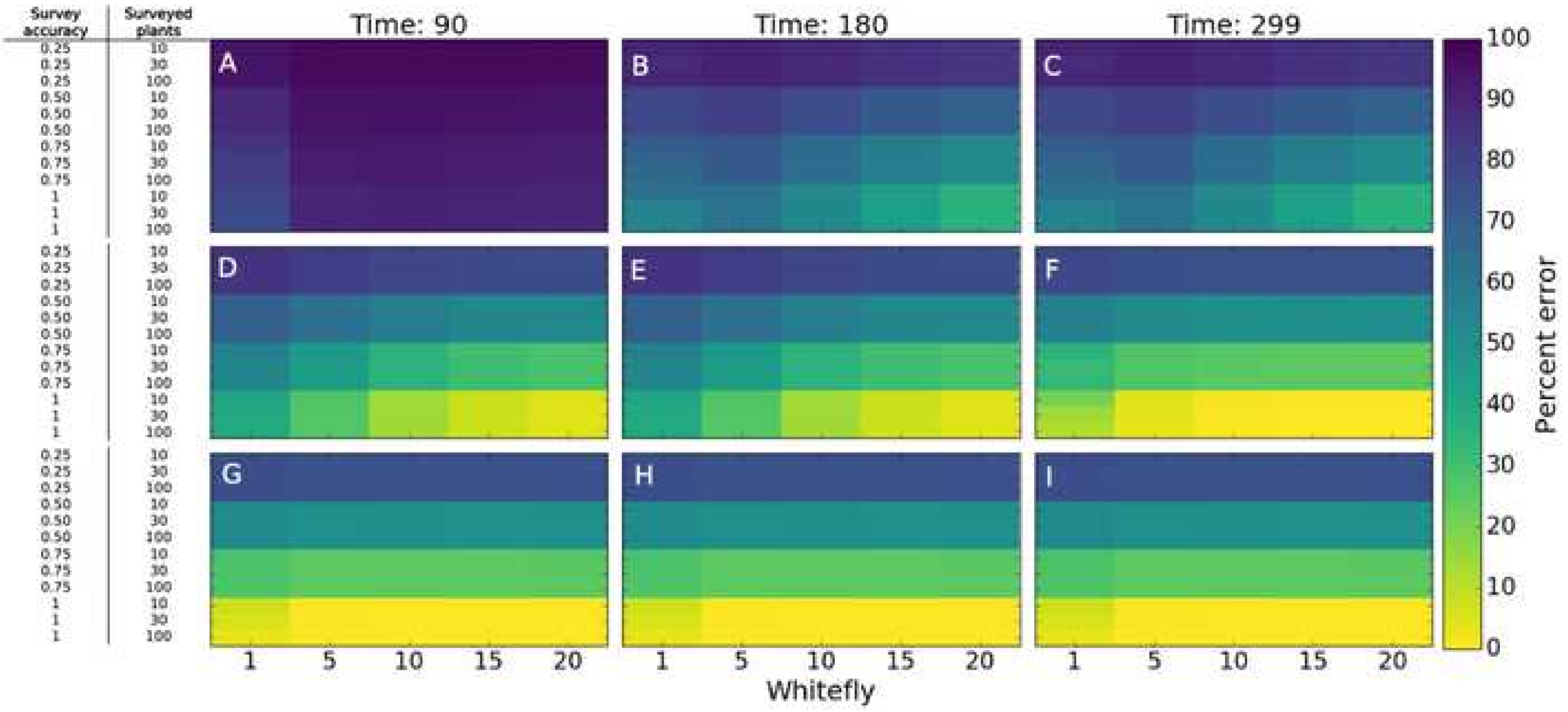

Click here to download Figure Fig7.tif

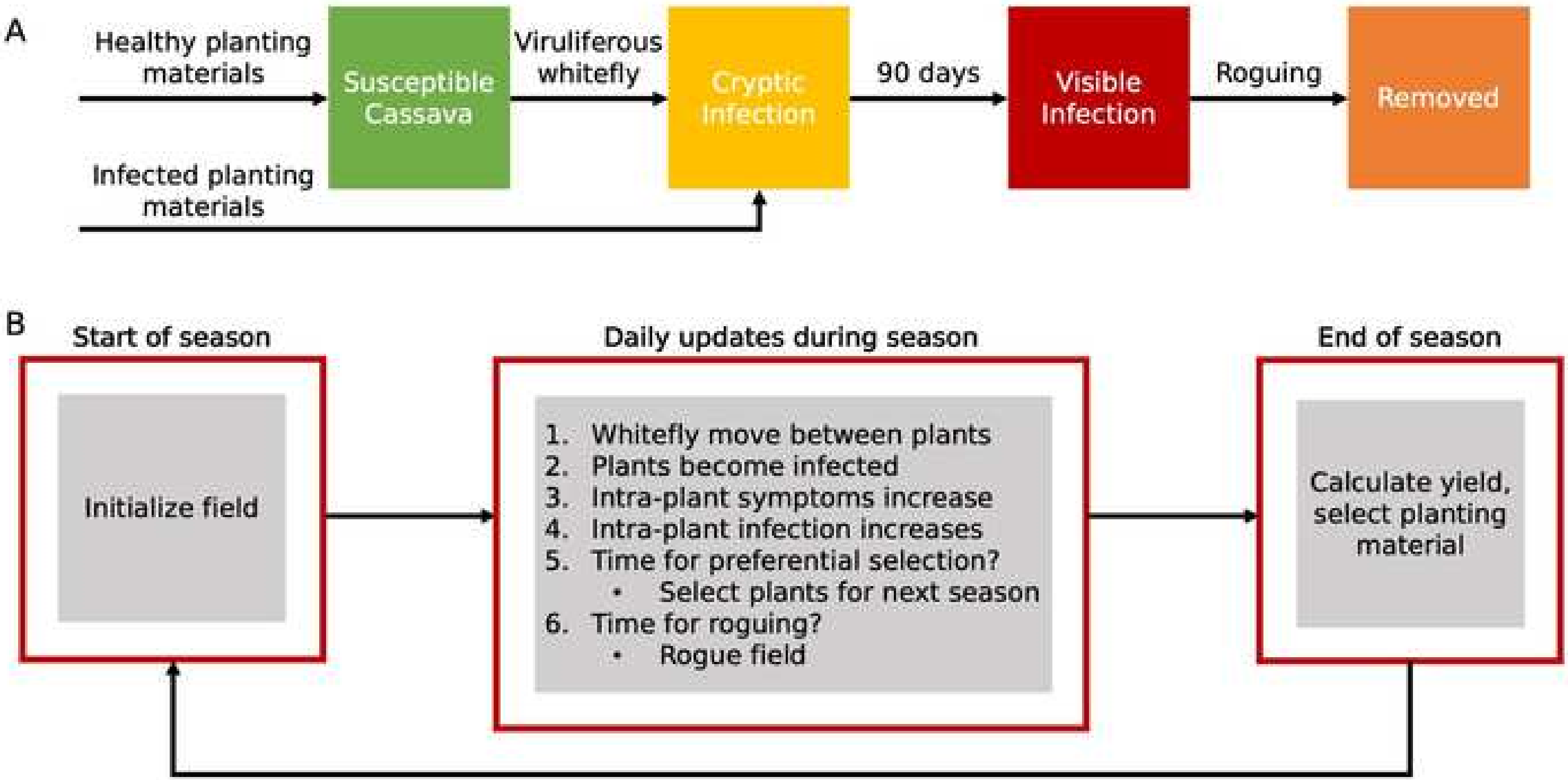

Click here to access/download
Supporting Information
FigS1.tif

Click here to access/download
Supporting Information
FigS2.tif

Click here to access/download
Supporting Information
FigS3.tif

Click here to access/download
Supporting Information
FigS5.tif

Click here to access/download
Supporting Information
FigS6.tif

Click here to access/download
Supporting Information
S4 Movie.mp4
