# Supplementary material for: Computational models to improve surveillance for cassava brown streak disease and minimize yield loss
Source: PLoS Comput Biol. 2020 Jul 2;16(7):e1007823. doi: 10.1371/journal.pcbi.1007823 (PMC7331984; doi:10.1371/journal.pcbi.1007823)
Supplement: Attachment — Submitted filename: Review of PCOMPBIOL.docx [file pcbi.1007823.s008.docx]

Review of PCOMPBIOL-D-19-01812, “Computational models to improve surveillance for cassava brown streak disease and minimize yield loss”

**General appraisal**

This is a sound and scientifically valuable paper which adds to our understanding of the management options of vectored diseases, and in particular of CBSD in Africa. Additionally, this paper provides options to optimise the surveillance for the detection of CBSD infected fields.

**General comments**

In this manuscript the authors develop a stochastic epidemiological model to assess different management options of CBSD in individual fields to minimise yield loss. Additionally, the authors examine surveillance strategies to successfully detect the presence of CBSD in infected fields.

Several Cassava Brown Streak Disease management interventions (roguing, preferential selection of planting material, deployment of clean seed and pesticide seed coating) are examined to evaluate their level of effectiveness on crop yield and disease status of individual cassava fields. These are simulated and analysed through a stochastic epidemiological model where interventions are evaluated individually and in combination in scenarios where whitefly numbers and disease pressure vary. The authors provide management options for different regions of West Africa depending on these variables.

The authors provide surveillance advice to detect the presence of the disease more accurately in infected fields but conclude that accurately predicting within-field prevalence is difficult.

The paper is well-written and accessible to non-specialists, but the format is somewhat difficult to follow. I would prefer to see the methods section before the results as the reader needs to understand the model before making sense of the results, but I also understand that this is the format established by PLOSCOMPBIOL.

My main comment is that the model and methodology need a lot more explanation for someone to be able to reproduce the results presented by the authors. The method section is mostly descriptive with the additional Figure 7 explaining the schematics of the model and the lines 96-108 explaining what kind of model has been developed. In order for the reader to follow and understand the results, the reader has to understand the model and only brief explanations of this appear throughout the text. The authors should add a section where the model is included and explained thoroughly. Or are the authors considering uploading the model and / or the code as supplementary material? An annotated code could at least make up for lack of details in the paper.

**Specific comments**

1. Authors mention that tolerant varieties showing more foliar symptoms may be more suitable for planting when cultural management such as roguing takes place in a field. This is generally true when resistance reduces the transmission of infection. However, the authors should mention that choosing the right tolerant or resistant varieties is crucial for the long-term management of CBSD as some tolerant varieties may increase the selection for more virulent virus strains.
2. Figure S1 is mentioned very frequently in the text. The authors should consider placing it in the main text as it is important to provide the necessary context for the reader to understand the whole study.
3. Detailed comments are provided in an annotated PDF version of the submitted manuscript.
