# Supplementary material for: Computational models to improve surveillance for cassava brown streak disease and minimize yield loss
Source: PLoS Comput Biol. 2020 Jul 2;16(7):e1007823. doi: 10.1371/journal.pcbi.1007823 (PMC7331984; doi:10.1371/journal.pcbi.1007823)
Supplement: Attachment — Submitted filename: Response to Reviewers.docx [file pcbi.1007823.s010.docx]

**Reviewer 1**

- line 241-247: I found that stating "Increasing the roguing accuracy from low to high also led to a large improvement in the average number of cuttings generated.." slightly misleading. Figure 4 shows modest increases, and the trends in both look to be the same. Looking at the scale, which reflects that increases are by 10k, there isn't a benchmark for me to know if that is indeed a large increase, e.g. if 6000 cuttings creates 1 full additional field, the gains seem modest, unless I have missed something. I would recommend that presenting the information with an appropriate relative scale.
  - ***Response*** It is difficult to select a relative scale for the importance an arbitrary number of clean seed cuttings because there is so much potential variability with the number of cuttings an individual farmer would buy and the frequency that they would purchase clean seed. Instead of changing figure 4, we have added an additional paragraph to this section with an explicit example of calculating the change in the number of cuttings produced and an estimate of the number of farmers a single field could support.
  - ***Action*** We have added the following paragraph to the manuscript:

“The clean seed requirements for a region varies depending on the frequency that farmers introduce clean seed to their fields and the percentage of the field that is to be replanted. A single clean seed multiplication field could support up to 1800 farmers using low intensity clean seed (replanting 10% of a field every three years) or up to 750 farmers using high intensity clean seed (replanting 25% of a field every two years) assuming that none of the plants in the field become infected (Table 2). This means that a change of 20,000 cuttings produced, for example by using low intensity roguing and pesticide in an area with moderately abundant whitefly, would translate to a clean seed producer being able to support an additional 100 farmers using low intensity clean seed or 27 using high intensity.”

- line 266-269: I find that the sentence does not read well and could be better written.
  - ***Action*** We have reworded the sentence to make it clearer. It now reads:

“When 5% of a field is infected with CBSD at the beginning of a growing season, surveying at least 30 plants per field results in a high probability of detecting infection. Conducting foliar surveys later in the growing season increases the likelihood of detecting disease, regardless of survey accuracy and whitefly abundance (Fig. 5).”

- line 297: "...combination of survey variables (surveyor accuracy number of plants surveyed)..." It seems to me there is a missing word within the parentheses.
  - ***Action*** We have fixed the typo by adding the word “and”. The phrase now reads: “the y-axis is a different combination of survey variables (surveyor accuracy and number of plants surveyed).”
- line 494: is this a negative exponential kernel? The kernel is not described, unless I have missed this. I would like to see this.
  - ***Action*** We have added the specific equation we are using for the kernel to the previous sentence to clarify that we’re using a negative exponential kernel.
- For the authors interest in increasing surveillance I would like to bring their attention to the app called Plantix if they have not heard of this before. https://plantix.net/en/ It has been a very successful diagnostic framework within India for pests and diseases of agricrops. Attempts are under way to use data collected from Plantix to help with detection and prevention of crop losses from pests and diseases. I feel that discussions with the Plantix team would benefit future work for CBSD in West Africa.
  - ***Response*** We thank the reviewer for suggesting Plantix. We have interacted with various groups like PlantVillage that provide diagnostics in the form of image based disease diagnostic apps.

**Reviewer 2:**

- Authors mention that tolerant varieties showing more foliar symptoms may be more suitable for planting when cultural management such as roguing takes place in a field. This is generally true when resistance reduces the transmission of infection. However, the authors should mention that choosing the right tolerant or resistant varieties is crucial for the long-term management of CBSD as some tolerant varieties may increase the selection for more virulent virus strains.
  - ***Action*** We have expanded our discussion about choosing characteristics of improved varieties including the risks from asymptomatic multiplication of virus in tolerant plants in the Discussion section.
- Figure S1 is mentioned very frequently in the text. The authors should consider placing it in the main text as it is important to provide the necessary context for the reader to understand the whole study.
  - ***Response*** We thank the reviewer for the suggestion which we have considered carefully. On balance, we think there is a risk that bringing the complete results including ineffective intervention may dilute one of the principal messages of the paper which is to identify the most successful interventions (i.e. clean seed and all interventions combined). We therefore prefer to retain the full results in figure S1 in the supplement.
  - ***Action*** No change after careful consideration.
- line 68: Make clear that tolerant is not equal to resistant. Tolerant may lead to evolution of resistance in the medium/long term
  - ***Action*** We have clarified our definition of tolerant varieties here and added greater depth about tolerant vs resistant varieties in the discussion. The sentence now reads: “Introducing virus-free planting material (known as ‘clean seed’) or tolerant varieties with reduced disease symptoms can also be used to improve yield. Tolerance, however, has the disadvantage of maintaining high levels of virus titer in infected plants without displaying symptoms of infection. ”
- Line 96: These two really need to be explained in methods. How do you do the modelling explicitly, both for the surveillance and for the epidemiology and management application?
  - ***Response*** We thank the reviewer for pointing out the needs for more clarity in the methods.
  - ***Action*** We have expanded the methods section with more detail about how the model works. Additionally, we have created a GitHub repository with an annotated version of the model code and example input files, which is now referenced in the methods section.
- Line 126: How many plants per field do you have? Will your results depend on the field size?
  - ***Response*** The field is large with respect to the dispersal kernel, so there should be minimal edge effects and local dynamics should be the same in a larger field. Also, we have selected a field size that is representative of small holder cassava fields to reduce the amount of scaling that will be needed to apply the results.
  - ***Action*** We have added the number of plants per field and the shape of the field to the preceding sentence, which is typical of smallholder cassava fields, We have also noted that the field size is large relative to the dispersal kernel.
- Line 148: achieve or reach would be a better word
  - ***Action*** We have changed the sentence to use the word achieve.
- Line 154: will this then mean that in general it is better to do nothing instead of roguing? Unless it is for the production of clean seed as shown in Figure 4 ?
  - ***Response*** For an individual farmer, roguing can increase yield with low whitefly and low starting infection (figure S1), but in general the simulations suggest that it’s better to do nothing than to rogue. However, it is better for the region to reduce the overall level of disease through roguing because this will reduce the spread of the disease to neighboring fields.
  - ***Action*** We have added a more thorough overview of this point to the discussion section.
- Line 165: I do not fully understand this figure. Is the first "drop" (approximately at 1/9th of each season, Fig. 1) corresponding to yield decrease due to disease symptoms development? Or why do we have this jumps? I assume that the other two drops in each season correspond to the surveying roguing (approximately at 1/3 and 2/3 of each season). This should be made clearer. How does this look when surveys are done a little later, e.g. when symptoms manifest more clearly?
  - ***Response*** In the model, new plants are unable to be infected for the first 30 days in the growing season (which corresponds with when the first leaves emerge and whitefly are able to infect plants). The yield gradually decreases as new plants become infected and the two larger drops correspond to roguing. Conducting surveys later in the season would presumably increase the effectiveness of roguing; however, in practice foliar surveys cannot be conducted after six months because the highly symptomatic leaves fall off, and the plants are too tall. The specific times used for roguing, preferential selection, and disease surveillance correspond to protocols currently in use.
  - ***Action*** We have added some extra description to the fig 1 legend to clarify the relationship between yield and timing of infection and roguing
- Line 179: but only for low whitefly density and high control intensity. In all other cases, it doesn't make a difference. For example low intensity is only useful when there is a very low whitefly density (fig s1). A big conclusion would be that if there is low whitefly density there are options for management. When whitefly is abundant, the only way to increase yield using clean seed is high intensity control

***Response*** We thank the reviewer here and elsewhere for the careful reading of the paper which has helped the authors to improve the clarity of the paper.

***Action*** We have added a new sentence to clarify that high intensity clean seed is needed in many circumstances to improve yield. Additionally, we have added to the discussion to emphasize how much whitefly abundance affects options for management interventions.

- Line 182: How much? It looks from the figure up to 60% in high clean seed and 40% in low clean seed!
  - ***Action*** We have added the exact yield improvement for a specific set of starting conditions to better illustrate the difference between random and block planting.
- Line 219a: According to Fig S1 I would say completely ineffective
  - ***Action*** Change made. Sentence now reads: “In regions with moderate whitefly but low disease pressure, roguing and preferential selection become ineffective,”
- Line 219b: not so much at low intensity
  - ***Action*** Change made. Sentence now reads: “but clean seed and pesticide coating are somewhat effective at low intensity and more so at high intensities”
- Line 221: I think this should be figure 2
  - ***Action*** Thank you for noticing this, we have corrected the typo.
- Line 272: I don't see how this figure is related to the probability of detecting CBSD. Can you explain? Also, if this is related to figure 5, it is stated above that 5% of the field is infected. I am a little confused with this sentence here.
  - ***Response*** Apologies for the confusion caused by a typo.
  - ***Action*** We have corrected the typo to correctly reference Fig S3, which is essentially analogous to Figure 5 but starting with only one infected plant.
- Line 279: Also mention here that 5% of the plants of the field are infected.
  - ***Action*** We have clarified in the figure title that the initial infection is 5%. The title now reads: “The probability of detecting infection in a field with an initial within-field incidence of 5%.”
- Line 301: It is interesting to see that the differences between starting infections of 20% and 50% for all accuracies an high number of whitefly do not make much of a difference in the estimated prevalence. What does this mean?
  - ***Response*** When the disease prevalence is in the region of 20-50%, the infection spreads fairly quickly within the field and the infection tends to be more uniformly distributed. Whereas with a starting incidence of 5%, the infection is present in sparser, more discrete clumps and the two survey transects tend to be much less representative.

- Line 361: This is presumably due to higher disease prevalence, but wouldn't it be better to detect at lower prevalence? Most of the simulation results show that best control is obtained when there is only one diseased plant per field whereas control (in particular cultural one) fails as incidence increases. I know this is what simulations show, but may be worth mentioning that as probability of detecting disease increases, the ability to manage disease through certain methods decreases.
  - ***Response*** Detecting the disease earlier in the season would mean surveying many more plants per field (Fig 5 suggests closer to 100 plants would be required), which would reduce the total number of fields that could be surveyed. Also in practice, there’s no immediate response to detection of CBSD, so it doesn’t matter when during a growing season the disease is detected.
- Line 377: when incidence has reached more than 20%, but for low incidence it is almost impossible to detect CBSD. Even for 100 accuracy and 100 plants the probability of detection is below 50% for all seasons and whitefly numbers. What does this say? Maybe add a reference or a paragraph outlining what happens when nothing is detected.
  - ***Response*** The probability of detecting any infection in a field is shown in figure 5, and with the right survey protocol, CBSD can be reliably detected when the starting within-field incidence is at least 5%. The reason that the percent error for estimating within-field prevalence is so high in general, and particular in fields with a low starting infection has to do with the non-uniformity of infected plants within a field. Presumably by surveying more plants this could be overcome, but with the current approaches, surveying more than 100 plants/field is not practical.
- Line 378: Delete hyphen
  - ***Response*** We are referring specifically to the prevalence within a single field rather than regional prevalence level, so we think it is appropriate to use a hyphen here.
- Line 397: Immediately after
  - ***Action*** We have changed the wording. The sentence now reads: “We also assume that immediately after a plant becomes infected there is zero chance for emigrating whitefly to carry the virus, and that the infectiousness of a plant increases linearly until it becomes maximally infectious after 90 days, reflecting the experimental results of Rwegasira and Rey [32] (Table 1, Fig. 7A).”
- Line 433: Mention it in the text
  - ***Action*** We have added the plant spacing to the text as well as clarifying both the total number of plants and the field dimensions in the table
- Line 478: This means that accuracy will depend on both, cultivar and disease incidence. What is the exact calculation? As with model can it be made explicit in the text please?
  - ***Action*** We have added the equation that is being used to determine if a given infected plant is identified and clarified how we model surveys for different cultivars. The probability of detecting if a plant is infected depends only on the symptoms and the survey accuracy.
- Line 491: What is the justification for this? Maybe it is just for simplicity, maybe it is biologically sound, but please clarify.
  - ***Response*** If the whitefly abundance fluctuates, the results should be between the values (i.e. bounds) we report for the two constant values, and modelling the effect of whitefly dynamics is beyond the scope of the current paper. The CBSD spread data we are using from Katono et al. (2015) include whitefly prevalence data from each of the different field sites and all three locations have different whitefly dynamics. Because there are not enough data from other sources about whitefly dynamics within a growth season, we have chosen for simplicity to use a constant value.
  - ***Action*** We have added a brief justification to the methods section.

**General reviewer comments**

- Have all data underlying the figures and results presented in the manuscript been provided?
  - ***Response*** All of the data used in the paper have been previously published.
  - ***Action*** We have clarified the data subsection in the Methods to explicitly list where each of the different types of data (disease progression time courses/monthly whitefly counts, cassava density in sub Saharan Africa, whitefly abundance in sub Saharan Africa, and CBSD detection in various countries) have been published.
